# Expression of a Cryptic Secondary Sigma Factor Gene Unveils Natural Competence for DNA Transformation in *Staphylococcus aureus*

**DOI:** 10.1371/journal.ppat.1003003

**Published:** 2012-11-01

**Authors:** Kazuya Morikawa, Aya J. Takemura, Yumiko Inose, Melody Tsai, Le Thuy Nguyen Thi, Toshiko Ohta, Tarek Msadek

**Affiliations:** 1 University of Tsukuba, Division of Biomedical Science, Faculty of Medicine, Tsukuba, Japan; 2 Institut Pasteur, Biology of Gram Positive Pathogens, Department of Microbiology, Paris, France; 3 CNRS ERL 3526, Paris, France; University of Tubingen, Germany

## Abstract

It has long been a question whether *Staphylococcus aureus*, a major human pathogen, is able to develop natural competence for transformation by DNA. We previously showed that a novel staphylococcal secondary sigma factor, SigH, was a likely key component for competence development, but the corresponding gene appeared to be cryptic as its expression could not be detected during growth under standard laboratory conditions. Here, we have uncovered two distinct mechanisms allowing activation of SigH production in a minor fraction of the bacterial cell population. The first is a chromosomal gene duplication rearrangement occurring spontaneously at a low frequency [≤10^−5^], generating expression of a new chimeric *sigH* gene. The second involves post-transcriptional regulation through an upstream inverted repeat sequence, effectively suppressing expression of the *sigH* gene. Importantly, we have demonstrated for the first time that *S*. *aureus* cells producing active SigH become competent for transformation by plasmid or chromosomal DNA, which requires the expression of SigH-controlled competence genes. Additionally, using DNA from the N315 MRSA strain, we successfully transferred the full length SCC*mec*II element through natural transformation to a methicillin-sensitive strain, conferring methicillin resistance to the resulting *S. aureus* transformants. Taken together, we propose a unique model for staphylococcal competence regulation by SigH that could help explain the acquisition of antibiotic resistance genes through horizontal gene transfer in this important pathogen.

## Author Summary

*Staphylococcus aureus* is a major human pathogen responsible for a broad spectrum of infections, emphasized by the emergence of multiple antibiotic-resistant strains with up to 60% of strains worldwide resistant to methicillin (Methicillin Resistant *Staphylococcus aureus* or MRSA). Indeed, MRSA-related infections are now one of the leading causes of death in the USA, highlighting the growing threat this bacterium poses to human health. Many bacteria have the ability to acquire novel genetic characteristics, including antibiotic resistance, through the uptake of extracellular DNA, a phenomenon known as natural genetic transformation or competence. We have shown that the SigH staphylococcal sigma factor is a likely key component for competence development, but that its gene is not expressed under standard laboratory conditions. Here, we have uncovered two distinct mechanisms allowing activation of SigH production in *S*. *aureus*: a chromosomal gene duplication rearrangement and post-transcriptional regulation through an upstream inverted repeat sequence. Importantly, we have demonstrated for the first time that *S*. *aureus* cells producing active SigH become competent for natural transformation by plasmid or chromosomal DNA, and we were able to confer methicillin resistance to a methicillin-sensitive strain by transformation with chromosomal DNA. SigH-dependent competence development in *S*. *aureus* could help explain the acquisition of antibiotic resistance genes and the rise of the so-called “superbug."

## Introduction

*Staphylococcus aureus*, first discovered and described over 130 years ago [1], [2], belongs to the low G+C % Gram-positive Bacilli class of Firmicutes that also includes *Bacillus subtilis* and *Listeria monocytogenes*. A commensal bacterium, often colonizing mammalian nasal cavities [3], *S. aureus* is also a major human pathogen causing a broad spectrum of infections ranging from food poisoning and superficial skin abscesses to more serious diseases such as pneumonia, meningitis, osteomyelitis, septicemia, toxic shock syndrome and sepsis [4]. It has acquired resistance to a wide variety of antibiotics [5], [6], and methicillin-resistant strains (MRSA), the most common cause of nosocomial infections, are now spreading into the community [7].

The *S. aureus* genome contains several mobile genetic elements such as transposons, bacteriophages, insertion sequences, pathogenicity islands and a staphylococcal cassette chromosome (SCC) [8], [9], which carry many of the toxin and antibiotic resistance genes. This indicates that horizontal gene transfer (HGT), occurring in bacteria through multiple mechanisms [10], must play a critical role in the evolution of this major human pathogen.

Conjugation in *S. aureus* requires a series of *tra* genes or conjugative plasmids, which are only found in certain isolates [11]. Most *S. aureus* strains are lysogenized with temperate phages, which can enter a lytic cycle that leads to generalized transduction when host DNA is mispackaged into some phages and transferred to a recipient cell upon the following infection. In addition, unusual phage-like infectious particles are involved in the efficient transfer of staphylococcal pathogenicity islands [12].

Natural genetic competence for transformation involves the binding and uptake of extracellular DNA [13]. Following a publication in 1972 reporting the existence of a transformation-like phenomenon in *S*. *aureus* [14], numerous reports investigating this process appeared over the ensuing decade. It was finally shown that this was in fact not natural genetic competence, but a type of HGT that requires contaminating phage tail fragments in the DNA preparation, which bind to the host cell and allow entry of DNA [15]. Thus, to date, *bona fide* DNA-mediated transformation of *S*. *aureus* by natural genetic competence has not yet been detected and remains something of a “holy grail".

Despite this fact, orthologues of most of the competence genes encoding the DNA uptake machinery, such as the *comG* and *comE* operons [13], [16], are present and conserved in staphylococcal genomes [17], [18] (See Supplementary Material Figure S1). This has also been reported for several other supposedly non-competent bacterial species, such as *Listeria monocytogenes* or *Lactococcus lactis* [19]–[21], indicating that we do not yet fully understand the specific conditions required for competence development in these bacteria and that they may in fact be able to become competent.

We previously identified a novel alternative sigma factor in *Staphylococcus aureus*, SigH, and demonstrated that it associates with core RNA polymerase to specifically transcribe the *comG* and *comE* competence operon orthologues in *S*. *aureus* [18]. In addition to the primary vegetative sigma factor SigA [22], *S. aureus* has at least three alternative sigma factors: SigB [23]–[25], SigH [18], and SigS [26]. SigB is responsible for a variety of stress responses and is activated by a partner-switching regulatory mechanism [27], while the function of SigS remains elusive [26].

Staphylococcal SigH has a unique evolutionary characteristic in that it shares exceptionally low sequence similarity between different bacterial species [18], [28]. Phylogenetic studies indicate that it belongs to a large group also including SigH of *Bacillus subtilis* [29], and ComX of *Streptococcu*s *pneumoniae* [30]. These related sigma factors are widely distributed among *Firmicutes*, with diverse physiological roles: in *B. subtilis*, SigH (Spo0H) is required for transcription of several early sporulation genes [31], [32] whereas in *S. pneumoniae*, ComX (SigX) directs the expression of late genetic competence genes in response to a peptide quorum-sensing regulatory pathway [30], [33], [34].

In *S*. *aureus* however, the *sigH* gene appears to be cryptic since its expression could not be detected under standard laboratory culture conditions, although its artificial overexpression *in vivo* induces expression of the *comE* and *comG* operons [18]. Because true bacterial cryptic genes are more akin to pseudogenes, likely to be lost through “use it or lose it" evolutionary constraints [35]–[37], the high conservation within *S*. *aureus* strains of SigH and the competence gene orthologues it controls suggested they must be expressed and play a role under certain specific growth conditions.

In this study, we wished to gain insight into staphylococcal competence development by identifying conditions where the SigH secondary sigma factor is active. We report the existence of two distinct mechanisms allowing activation of SigH production in a minor fraction of the cell population and have successfully demonstrated for the first time that *S*. *aureus* cells producing active SigH become competent for transformation by DNA, in a manner dependent upon SigH-controlled competence genes. We propose a model for staphylococcal competence regulation, and discuss its evolutionary differences with the known systems in *B. subtilis* and *Streptococcus* species as well as the crucial role competence likely plays in acquisition of antibiotic resistance genes.

## Results

### SigH active cells can be isolated and stably maintained

**Figure 1:**
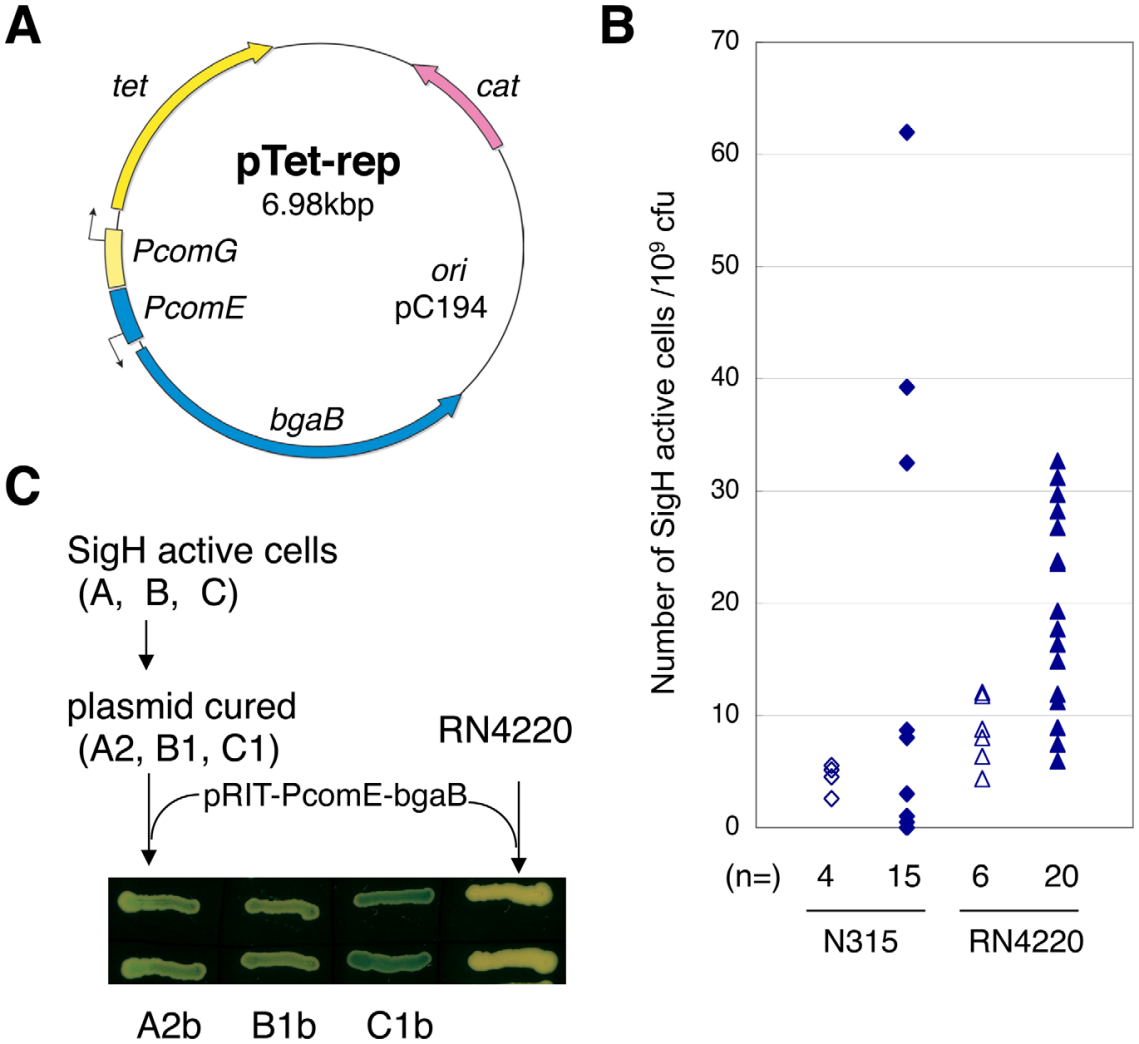
Positive selection for *Staphylococcus aureus* cells with active SigH. **A**) Positive selection tetracycline resistance (*tet*) reporter plasmid (pTet-rep). Reporter strains carrying pTet-rep were selected with 5 µg/ml tetracycline. *cat*: chloramphenicol resistance gene. *bgaB*: β-galactosidase gene. PcomG: promoter region of *comG* operon. PcomE: promoter region of *comE* operon. **B**) Fluctuation tests indicate that SigH activation occurs spontaneously. The y-axis represents the numbers of SigH active colonies detected from 10^9^ cfu. Cells were grown in drug-free TSB and then selected for tetracycline resistance. Open symbols: aliquots from a single flask. Closed symbols: independent cultures in separate test tubes. Diamonds: N315 pTet-rep, Triangles: RNtet-rep (RN4220 pTet-rep). **C**) SigH activity can be stably maintained through generations without selection pressure. pTet-rep was cured from SigH active cells, and pRIT-PcomE-bgaB was introduced. Cells were grown on TSA plates containing 100 µg/ml X-gal and 12.5 µg/ml chloramphenicol. Rare white cells (about 1%) that lost SigH activity were also observed (see text).

In order to isolate cells where SigH is produced and active, the pTet-rep positive selection double-reporter plasmid was constructed, using the *tet* tetracycline antibiotic resistance gene and the *bgaB* β-galactosidase gene from *Bacillus stearothermophilus* [38] (See Materials and Methods; Figure 1A). The SigH-dependent *comG* (*SA1374*) and *comE* (*SA1418*) promoters [18] were used to monitor SigH activity by creating transcriptional fusions with the *tet* and *bgaB* genes, respectively. Reporter strains carrying pTet-rep are sensitive to tetracycline and do not exhibit β-galactosidase activity, due to the absence of endogenous SigH activity in *S*. *aureus* under standard laboratory growth conditions. Cells in which SigH activity spontaneously occurs and is stably maintained for a number of generations are thus expected to give rise to tetracycline-resistant β-galactosidase positive colonies.

The pTet-rep plasmid was introduced into *S*. *aureus* strain RN4220, giving strain RNtet-rep, and stationary phase cultures were plated in the presence of 5 µg/ml tetracycline. The majority of the tetracycline resistant colonies were also β-galactosidase positive, and using this approach we obtained SigH active cells from all *S*. *aureus* strain backgrounds tested (N315, RN4220, 8325-4 and MRSA408) with frequencies ranging from ca. 10^−5^∼<10^−9^ depending on the experiment (See Supplementary Material Figure S2). We measured the frequency fluctuation using the classical Luria-Delbrück test [39] (Figure 1B). When the reporter strain was cultured in a single flask, aliquots gave rise to similar numbers of SigH active cells. In contrast, when grown as multiple independent cultures, the number of SigH active cells varied (Figure 1B and Supplementary Material Figure S2). These variations suggest that SigH activation results from a spontaneous event, rather than artificial induction by the presence of tetracycline.

Three SigH active clones (designated A, B, and C) generated from strain RNtet-rep were chosen for further characterization of the stable SigH activation mechanism. Clones A and B exhibited similar β-galactosidase activities on X-gal plates, whereas clone C displayed higher levels. These SigH-positive phenotypes were not due to mutations in the reporter plasmids (Figure 1C). Indeed, the plasmids were cured from clones A, B, and C, to generate strains A2, B1, and C1, and a *comE*'-*bgaB* transcriptional fusion reporter plasmid, pRIT-PcomE-bgaB, was then introduced into these cells by electroporation (Figure 1C). The A2, B1, and C1 transformants were β-galactosidase-positive (designated A2b, B1b and C1b, respectively), whereas the parental RN4220 strain transformed with pRIT-PcomE-bgaB was not (Figure 1C), indicating that the SigH activity detected in clones A, B and C was stably maintained throughout these procedures. However, rare revertant white colonies emerged after overnight cultures of strains A2b, B1b and C1b, sometimes due to a deletion in the reporter plasmid but often to actual loss of SigH activity (see below). The frequency of cells where SigH activity was lost after a single overnight culture was ca. 10^−2^∼10^−3^. We observed no differences during growth in TSB medium between SigH active and SigH inactive cells.

### Increased SigH activity is associated with a reversible gene duplication through a chromosomal rearrangement event

**Figure 2:**
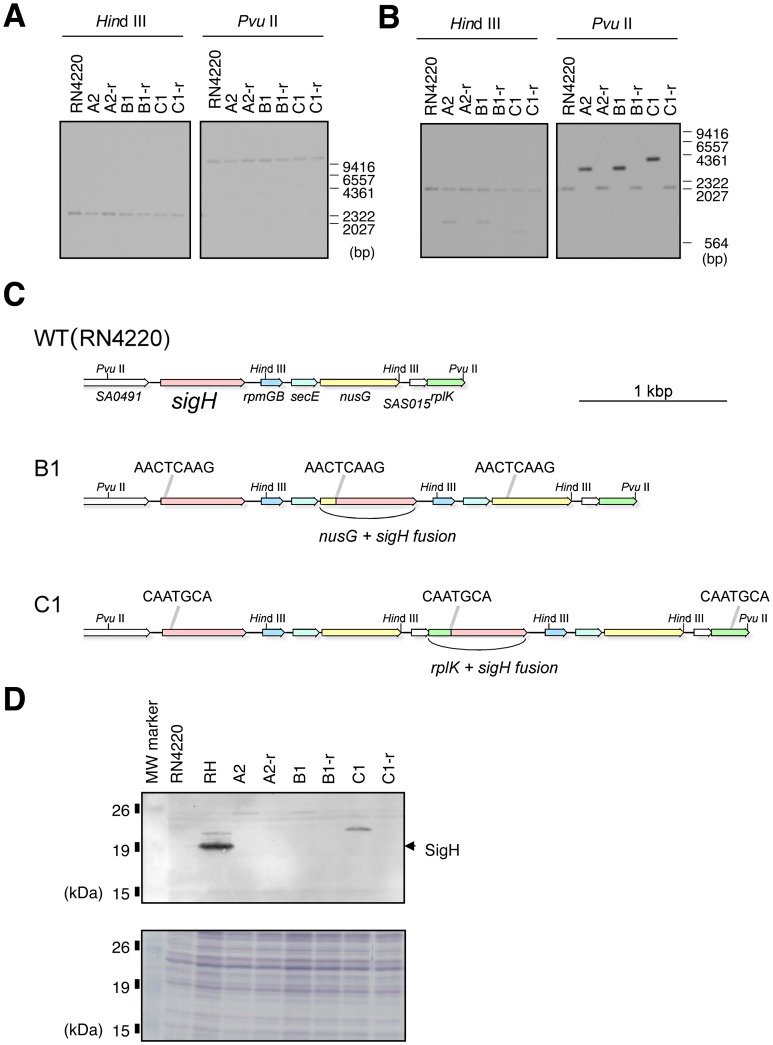
Activation of *sigH* expression involves a chromosomal rearrangement with a tandem gene duplication/fusion. **A**) and **B**) Southern blot analysis of the *sigH* locus for the parental strain (RN4220), SigH active mutants (A2, B1, C1) and revertants (A2-r, B1-r, C1-r). Genomic DNA was digested with *Hin*dIII or *Pvu*II. **A**) Blots were probed with a H1p-H2p PCR fragment (upstream region of *sigH*). **B**) Probed with H5p-SA0492Rp PCR fragment (*sigH* coding sequence). Positions of the DNA molecular mass marker (λ *Hin*dIII) are indicated in base pairs on the right. **C**) Schematic representations of the *sigH* loci of RN4220 and SigH active derivatives B1 and C1. Arrows represent coding sequences, and are colored by gene, e.g. red represents *sigH*, yellow represents *nusG* etc. The direct repeat sequences encompassing duplication units are indicated above the gene maps. These direct repeats originally exist in the RN4220 sequence, and the duplication event increases their number from two (in RN4220) to three (in B1/C1). Gene maps are illustrated to scale and the bar represents 1 kbp. **D**) Western blot analysis showing that SigH is expressed as a fusion protein encoded by the new chimeric genes. A2, B1, and C1 are SigH active derivatives. A2-r, B1-r, and C1-r are the revertants that have lost SigH activity. R: RN4220, RH: RN4220 + pRITsigH. The position of full-length native SigH (calculated molecular weight 23.0 kDa) is indicated by an arrow-head. Signals in B1 and C1 were observed at higher molecular weight positions, in agreement with the calculated molecular weights of the SigH chimeric proteins in strains B1 and C1 (28.3 kDa and 25.5 kDa, respectively). Sizes and positions of the Bench Mark pre-stained protein ladder (Promega) are indicated on the left. The lower panel is a loading control SDS-PAGE gel stained with Coomassie Brilliant Blue staining.

Southern blot analyses of the *sigH* locus were performed on genomic DNA of the A2, B1, and C1 strains displaying increased SigH activity (Figure 2A and 2B) as well as revertants from each clone that had lost SigH-dependent β-galactosidase activity after plasmid curing and introduction of the pRIT-PcomE-bgaB reporter plasmid (A2-r, B1-r, and C1-r are the revertants from A2b, B1b and C1b, respectively). When the upstream region of *sigH* was used as a probe (Figure 2A), there was no difference in pattern between the parental RN4220 and the SigH active strains. In contrast, when the *sigH* coding sequence was used as a probe (Figure 2B), an additional band (*Hin*dIII digest) or a slower migrating stronger signal (*Pvu*II digest) was detected for all of the SigH active mutants. This result suggests that the *sigH* gene is duplicated in the SigH active cells. Interestingly, the hybridization pattern for each of the revertants was identical to that of the RN4220 parental strain indicating that this is a fully reversible process. The duplication apparently occurs in close proximity to the *sigH* locus, because a stronger *sigH* signal was systematically observed in the *Pvu*II digests. This was confirmed by sequence analysis of the *sigH* region in strains B1 and C1.

### SigH is produced as a fusion protein encoded by a new chimeric gene generated by tandem duplication through a spontaneous chromosomal rearrangement

The nucleotide sequence of the entire *sigH* locus was determined for the B1 and C1 clones (Figure 2C). In the B1 clone, a 1210 bp region was found to be tandemly duplicated. The duplicated region was flanked by the 8 bp direct repeat sequence AACTCAAG. This duplication did not affect the structure of the original *sigH* gene, but instead generated a new chimeric gene with the downstream *nusG* coding sequence, with an intact copy of *nusG* still present. The chimeric *nusG*'-'*sigH* gene results from the precise in-frame fusion of codon 50 of the *nusG* coding sequence with codon 7 of *sigH*, such that the resulting chimeric NusG'-'SigH protein has its first seven amino acids replaced with the first 50 from NusG. In the case of clone C1, the 7 bp direct repeat sequence CAATGCA flanked a 1888 bp tandemly duplicated region, generating a new chimeric *rplK*'-'*sigH* gene, by fusing codon 42 of the downstream *rplK* gene with codon 16 of *sigH*, and retaining intact copies of *sigH* and *rplK* upstream and downstream of the duplication, respectively. This indicates that the duplication unit as well as the chimeric gene partner can vary, and that this process involves Short Junction (SJ) tandem duplication [40]. In both cases, the downstream gene's promoter, ribosome binding site and translational initiation codon drove expression of the resulting chimeric *sigH* gene. Western blot analyses indicated that the chimeric SigH proteins migrate in SDS-PAGE with a higher apparent molecular mass than native SigH, which is entirely consistent with the expected sizes of the chimeric proteins as deduced from the nucleotide sequence (SigH: 23 kDa, NusG'-'SigH: 28.3 kDa, RplK'-'SigH: 25.5 kDa; Figure 2D). Other types of SJ duplications leading to chimeric SigH proteins were also identified and are shown in Figure S3 (See Supplementary Material). The *sigH* locus nucleotide sequence of the B1-r and C1-r revertants that had lost SigH-dependent β-galactosidase activity was also determined, showing that the reversion had restored the native single copy *sigH* sequence following loss of the duplication.

### SigH is active in *S. aureus* under specific culture conditions

**Figure 3:**
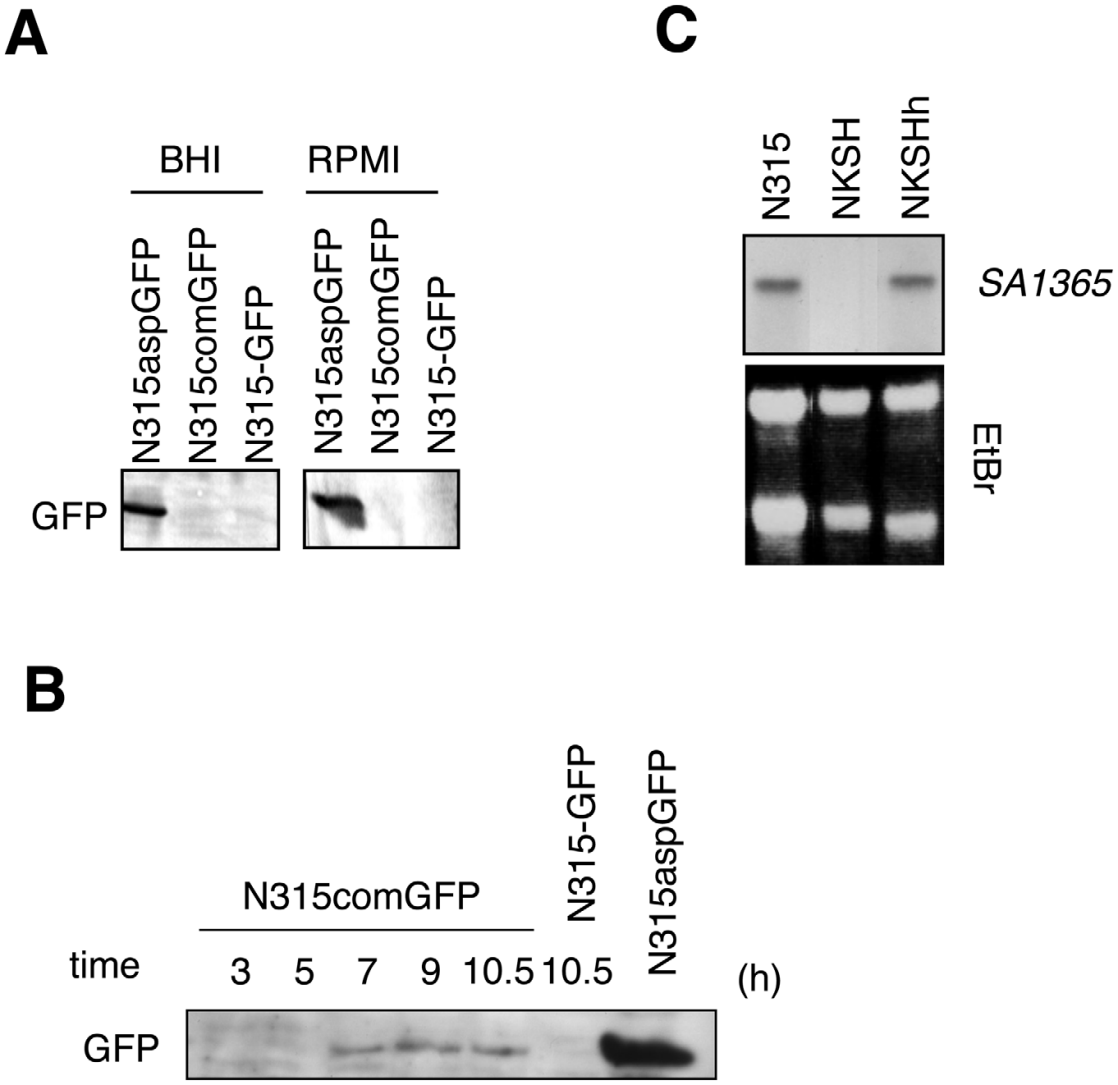
SigH is activated under specific growth conditions. **A**) Western blot analyses directed against GFP showing that SigH is not active in standard culture conditions. Strains N315aspGFP (*asp23* promoter), N315comGFP (*comG* promoter) and N315-GFP (promoter-less) were grown aerobically in BHI or RPMI 1640 medium. **B**) Western blot against GFP showing that SigH activity is induced in CS2 medium. Incubation times (hours) are indicated above the panel. Positive control cells of N315aspGFP were grown in TSB for 10.5 h. **C**) Northern blot analysis confirming SigH-dependent expression of *SA1365*, one of the *comG* operon genes. Strains N315, NKSH (*sigH* inactivation mutant), and NKSHh (complemented strain) were grown in anaerobic and static conditions in -GS medium.

In an attempt to identify specific growth conditions that could lead to production of active SigH in *S*. *aureus*, a reporter plasmid expressing GFP under the control of the *comG* promoter was constructed as described in Materials and Methods (pRIT-com-gfp). As negative and positive controls, we used pRIT-gfp (no promoter) and pRIT-asp-gfp where the σ^B^-dependent *asp23* promoter drives GFP expression. These plasmids were introduced into *S. aureus* strain N315 and GFP expression was examined by Western blot analysis. As shown in Figure 3A, *asp23* promoter activity was detected in standard laboratory media, such as BHI and RPMI 1640. In contrast, σ^H^-dependent expression was undetectable throughout growth under these conditions (Figure 3A). We saw no effect on SigH activity when cultures were exposed to different compounds or stress conditions, including hypertonic shock (1 M KCl or 2 M NaCl, for 20 min), heat shock (46°C for 20 min), freeze-thaw cycle, treatment with detergents (CHAPS, NP40, Tween 20, Triton X-100), or antibiotics (bacitracin, cefazolin, gentamycin, oxacillin, penicillin G, tetracycline, vancomycin at the Minimal Inhibitory Concentrations for 20 min).

In contrast, as shown in Figure 3B, we could clearly detect SigH-dependent production of GFP when cells were grown aerobically in a complete synthetic medium (CS2 medium; see Materials and Methods and Supplementary Material Table S1). Similar results were obtained when cells were grown anaerobically in other synthetic media (CS1 medium, -GS medium; see Materials and Methods and Supplementary Material Figure S4). The negative control strain without the *comG* promoter reporter (N315 pRIT-gfp) showed no GFP production under any of these conditions.

Northern blot analysis allowed us to show that the endogenous *SA1365* gene, the last gene of the σ^H^-dependent *comG* operon [18], is indeed actively transcribed under these SigH-activating growth conditions (Figure 3C). This transcription was lost in the NKSH *sigH* mutant strain, and restored in the NKSHh complemented strain expressing *sigH* from a multicopy plasmid (Figure 3C).

### SigH is only active in a minor fraction of the cell population

**Figure 4:**
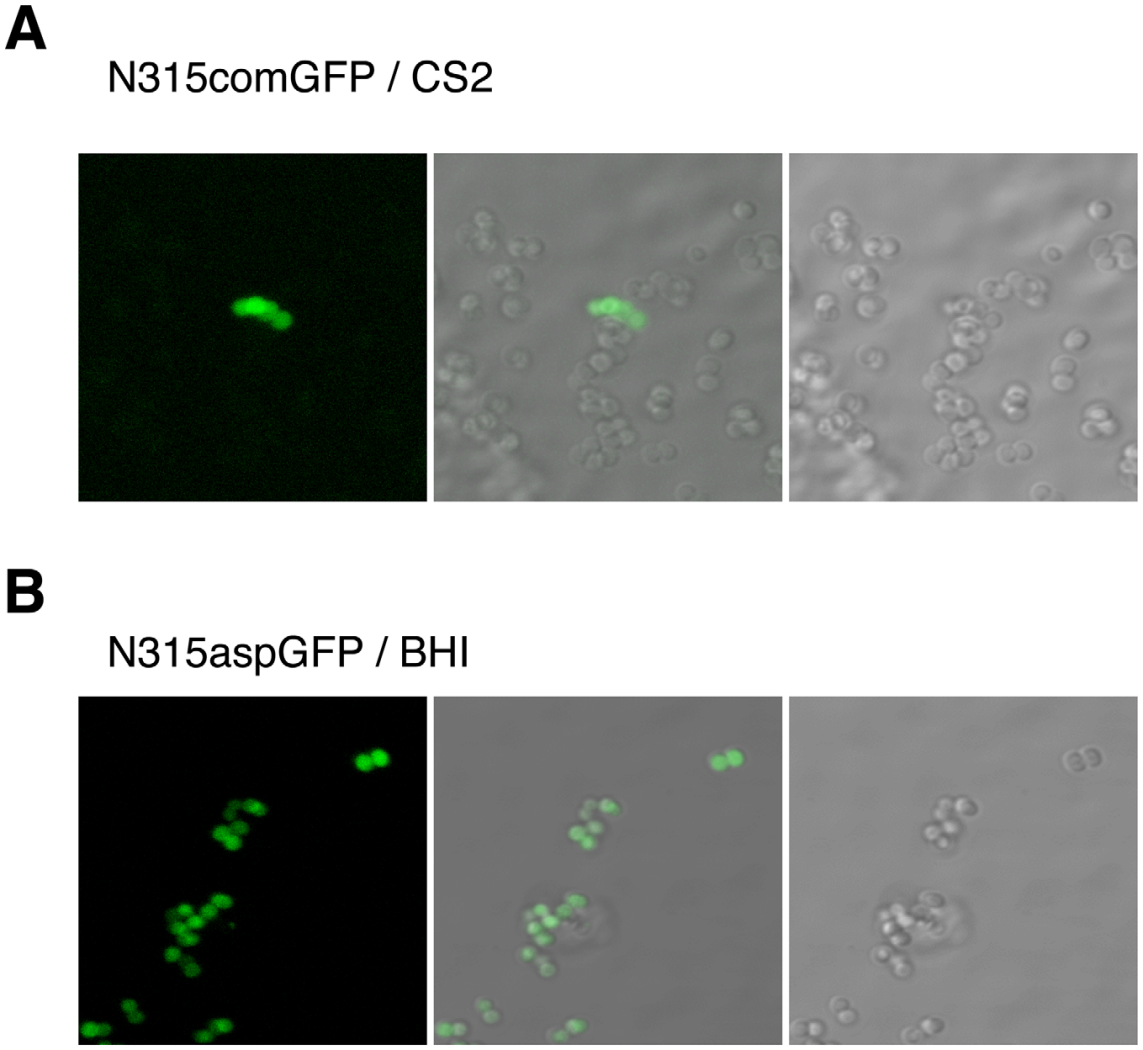
SigH is activated in a minor fraction of the cell population. Confocal microscopy reveals that SigH-dependent GFP expression is limited to a minor fraction of the cell population. In N315comGFP, the GFP-positive frequency was 1.6+−0.6% (mean ± SD, n = 10; Panel A), while 100% of N315aspGFP cells expressed GFP (Panel B). Left panels: Fluorescent GFP signals detected by confocal microscopy. Right panels: Phase contrast images. Middle: merged images. **A**) N315comGFP grown in CS2 medium. GFP expression is observed only in a minor fraction of the cell population. The maximum frequency of GFP-positive cells was about 1%. **B**) N315aspGFP grown in BHI medium. All cells express GFP.

Production of GFP in cells grown aerobically in CS2 medium was observed by confocal microscopy. Interestingly, only a small fraction of the cell population produced GFP when expression was driven from the SigH-dependent *comG* promoter (Figure 4A), with a frequency of 1.8±0.7% (at 7 h, n = 3). This is in clear contrast to the evenly distributed GFP expression from the SigB-dependent construct during growth in BHI (Figure 4B), whereas no GFP-positive cells were detected using the negative control strain (N315 pRIT-gfp).

### SigH activation occurs through multiple pathways

**Figure 5:**
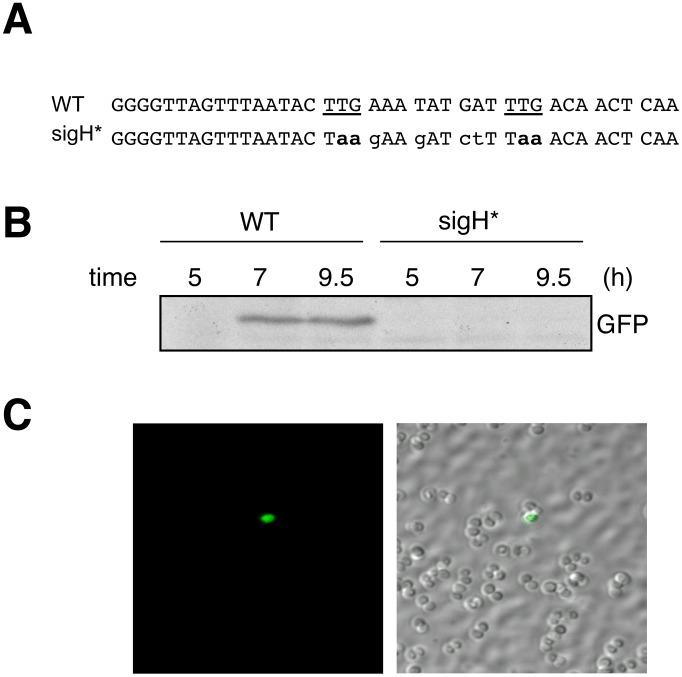
Two distinct mechanisms are responsible for SigH activation. The translation initiation codon of *sigH* is required for SigH activation in CS2 medium, but not for SJ-dependent SigH activation. **A**) Nucleotide sequence of the *sigH* translation initiation site region. The expected translation initiation codon, and the downstream in-frame TTG codon are underlined. Sequences for the wild type strain (N315ex; top), and the constructed mutant (N315ex-sigH*; bottom) are shown and the mutated residues are shown in lower case. **B**) Western blot analysis of SigH-dependent GFP production in WT (N315ex) and N315ex-sigH* carrying pMK3-com-gfp. Cells were grown in CS2 medium for the indicated times. No GFP signal was detected in the translation initiation codon mutant strain (N315ex-sigH*). **C**) Rare GFP expressing cells are still detectable in N315ex-sigH* carrying pMK3-com-gfp.

We tested whether the cells producing GFP observed in Figure 4A resulted from transient inducible SigH activation during growth in CS2 medium by a mechanism distinct from stable activation through SJ tandem duplication. We therefore introduced chromosomal mutations inactivating the two potential translation initiation codons of *sigH* (strain N315ex-sigH*; Figure 5A). These mutations should not affect SigH activation by SJ tandem duplication, which does not require the native *sigH* translation initiation site. The two reporter plasmids, pMKcomGFP and pTet-rep were then introduced into the mutant strain. Western blot analysis showed that GFP production during growth in CS2 medium was abolished in this strain (Figure 5B), whereas as expected, stable SigH activation (emergence of tet^R^ colonies) was observed in this mutant, but with only very few cells expressing GFP (Figure 5C). Thus, we conclude that two distinct mechanisms for activation of *S. aureus* SigH expression coexist: one requires the original translation initiation codon, and occurs stochastically during growth in chemically defined media, whereas the SJ-tandem duplication mechanism does not require the native *sigH* translation initiation sequences. The nucleotide sequence of the *sigH* locus from SigH active clones arising from the N315ex-sigH* mutant revealed SJ-tandem duplication events similar to those in the B1 and C1 clones (See Supplementary Material Figure S3).

### Post-transcriptional control of *sigH* expression involves the 5′-UTR

**Figure 6:**
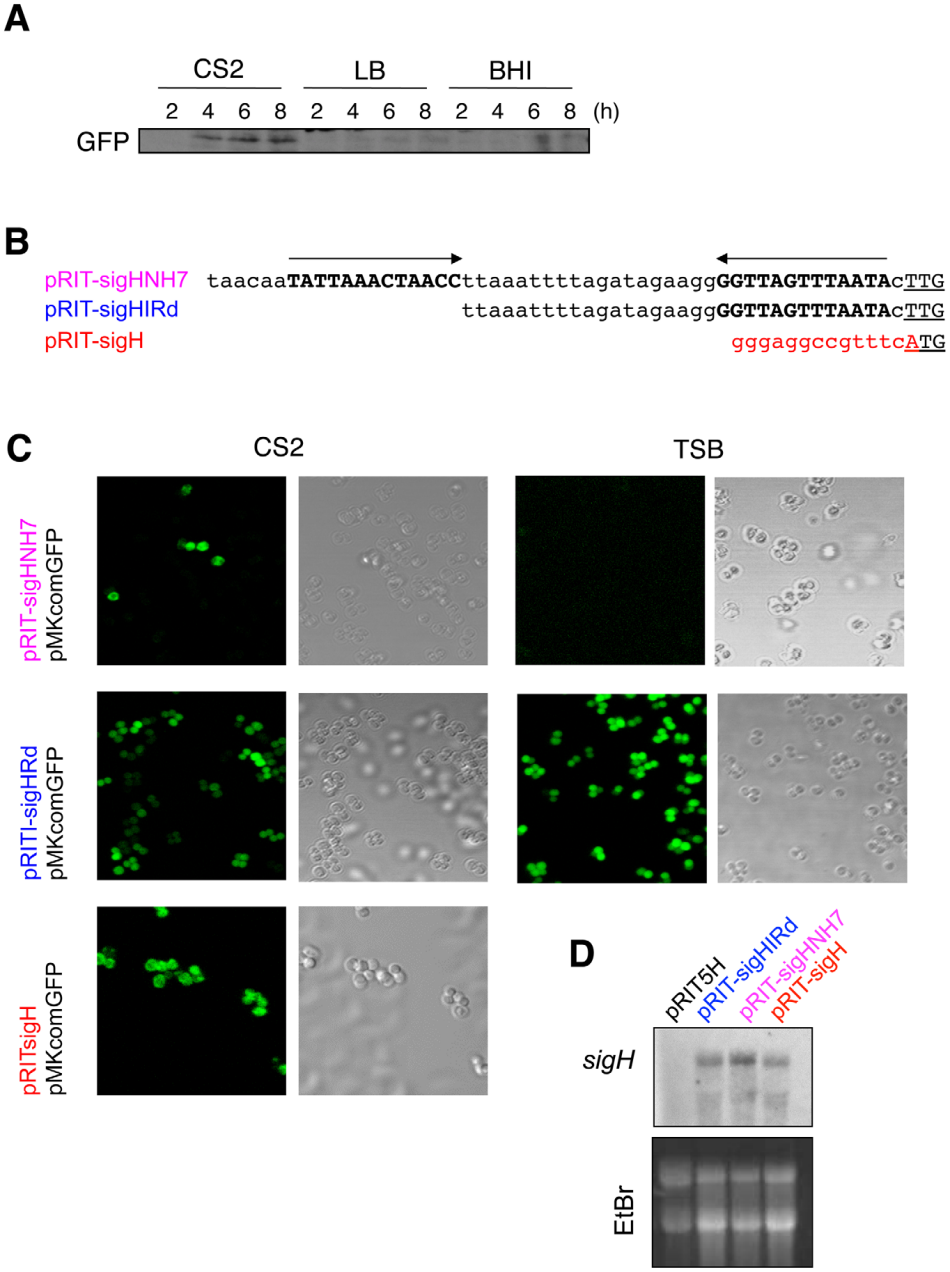
SigH activity is restricted to a minor fraction of the cell population through post-transcriptional regulation. **A**) Overexpression of *sigH* mRNA is not sufficient for SigH activation. N315ex carrying pMKcomGFP and pRIT-sigHNH7 was grown in CS2, LB, or BHI medium aerobically at 37°C for indicated time periods, and analyzed by Western blot. Only growth in CS2 medium led to GFP production. **B–C**) An inverted repeat sequence in the *sigH* 5′-UTR negatively controls its expression. **B**) Sequences of the 5′-UTR region in each *sigH*-expressing plasmid. Arrows show the 13 bp inverted repeat, and the translation initiation codon is underlined. PRIT-sigHNH7 carries the native nucleotide sequence. The inverted repeat sequence was partially deleted in pRIT-sigHIRd, whereas it was entirely removed and the SD sequence replaced with a consensus ribosome binding site in pRIT-sigH. **C**) Deletion of one half of the inverted repeat allows all cells to produce active SigH. N315ex derivatives carrying the plasmids indicated on the left were grown in CS2 or TSB at 37°C with shaking. Note that all cells of N315ex carrying pRIT-sigHIRd show GFP signals, even in TSB. **D**) Northern blot analysis confirmed the accumulation of *sigH* mRNA in cells carrying all of the different *sigH* expression plasmids. N315ex cells carrying the designated plasmid together with pMKcomGFP, were grown in TSB medium. Exponentially growing cells (OD_600_ = 0.5) were used for Northern blot analysis as described in Materials and Methods. The lower panel shows the EtBr stained agarose gel as a loading control.

Overexpression of *sigH* using a high-copy number plasmid (pRIT-sigHNH7) is not sufficient to induce SigH activity during growth in standard growth media such as BHI or LB (Figure 6A) [18]. In this plasmid, *sigH* is constitutively expressed from the *spa* promoter (staphylococcal protein A gene), indicating that increased transcription of *sigH* is not sufficient to produce active SigH. When this strain was grown in CS2 medium, accumulation of GFP was induced (Figure 6A), but again only in a minor fraction of the cell population (Figure 6C, top row), suggesting that some post-transcriptional regulatory mechanism limits SigH activation in the cell. Examination of the *sigH* DNA sequence revealed a perfect 13 base inverted repeat (IR), lying just upstream from the translation start codon, within the 5′ UTR region, likely sequestering the ribosome binding site within a stable stem-loop secondary structure (Figure 6B). We constructed a plasmid expressing *sigH*, with one half of the IR deleted (Figure 6B, pRIT-sigHIRd). Interestingly, all of the cells were now able to express σ^H^-dependent GFP (Figure 6C, middle row), both during growth in CS2 and TSB medium, indicating that this IR acts post-transcriptionally to negatively control SigH production. When we replaced the entire 5′ UTR region with a synthetic DNA sequence containing a consensus ribosome binding site (GGGAGG) and replacing the TTG initiation codon with ATG (plasmid pRIT-sigH, Figure 6B), we again observed σ^H^-dependent GFP expression in all of the cells (Figure 6C), confirming that *sigH* translation is the limiting step, in agreement with the results shown above from the SJ-tandem duplication, where the native translation initiation sequences were replaced in the resulting chimeric genes. Northern blot analysis confirmed that each of the three *sigH* expression plasmids allowed cells to accumulate similar amounts of *sigH* mRNA during growth in TSB (Figure 6D), confirming that post-transcriptional control of SigH production at the translational level is the limiting step.

### SigH production allows natural genetic transformation of *Staphylococcus aureus* cells

Efforts to detect natural genetic transformation in *S. aureus* have long been unsuccessful. Our findings indicate this is due at least in part to the fact that SigH production is restricted to a minor fraction of the cell population. Having established that growth in CS2 medium was conducive to production of active SigH, we used the strain overexpressing *sigH* from the pRIT-sigH plasmid and were able to establish for the first time a protocol through which reproducible natural transformation of *S. aureus* cells can be achieved (see Materials and Methods). Cells were grown in CS2 medium and incubated with pT181 plasmid DNA, and we obtained transformation efficiencies between 10^−8^∼10^−9^ (Table 1), and could show that all the transformants were the recipient strain carrying the intact plasmid (Supplementary Material Figure S5).

**Table 1:** Transformation frequencies of *S*. *aureus* strains.

	Recipient strain					
	N315h	N315Δ*comE*h	N315Δ*comG*h	N315	N315ex w/oφ	N315ex w/oφ
					pRIT-sigH	pRIT5H
Donor						
	4.0×10^−9^±3×10^−9^					
Plasmid DNA (10 µg pT181)	(n = 11)					
	ND	ND	ND	ND		
	(n = 1)	(n = 3)	(n = 3)	(n = 3)		
	9.0×10^−7^±6.6×10^−9^			2×10^−10^		
	(n = 12)			(n = 1)		
COL/pT181		ND	ND	ND		
		(n = 5)	(n = 4)	(n = 9)		
Plasmid DNA (10 µg pHY300)					1.5×10^−6^±6.3×10^−7^	ND
					(n = 3)	(n = 2)

Frequencies = number of transformants/cfu of recipient strain; mean ± SD.

ND: none detected.

In order to establish that natural genetic competence requires not only SigH, but the σ^H^-dependent competence gene orthologues as well, we introduced the SigH expression plasmid into strains lacking either the *comE* or *comG* operon (N315ΔcomEh and N315ΔcomGh, respectively). We carried out three independent experiments testing competence of these strains using plasmid pT181 and could not detect any transformants (less than 10^−10^; Table 1), indicating that, as expected, both the *comE* and *comG* operons are required for natural genetic competence in *S*. *aureus*, in addition to SigH.

In order to determine whether natural transformation also occurs using chromosomal DNA, we tested transfer of the MRSA SCC*mec* element from strain N315, which carries the *aadD* gene conferring kanamycin resistance, located 3.44 kb away from the *mecA* oxacillin resistance gene (See Supplementary Material Figure S6). The recipient strain, N315ex-h (Km^S^, OX^S^), is a N315 derivative that has lost the SCC*mec* region and carries the pRIT-sigH SigH production plasmid. Cells were grown in CS2 medium, incubated with purified N315 genomic DNA, and transformants were selected for kanamycin resistance (100 µg/ml). PCR analysis revealed that approximately 90% of the kanamycin resistant colonies contained the *aadD* gene (43/47), suggesting the remainder were spontaneous resistant mutants (Supplementary Material Figure S6A). The *aadD* transformation efficiency was 3.1×10^−8^, and the *mecA* gene was detected in 37 out of the 43 *aadD* positive clones, demonstrating that co-transformation using chromosomal DNA can be achieved for two distant markers. Long PCR analysis allowed us to show that these transformants in fact carry the entire 52 kbp SCC*mec*II element (Supplementary Material Figure S6B). The experiment was repeated twice and gave similar results.

We also tested whether HGT can occur between different cells. We found that the pT181 plasmid can be transferred from COL to N315h when these strains were co-cultured in CS2 medium (Table 1). The efficiencies varied depending on the experiments, likely due to the long co-cultivation time (8 hours). The median transformation efficiency from COL to N315h was 7.2×10^−8^ (min = 6.6×10^−9^, Q_1_ = 4.1×10^−8^, Q_3_ = 1.8×10^−7^, max = 9.0×10^−7^; n = 12). We could also detect rare HGT to wild type N315 (one transformant in 10 independent experiments).

As shown above, natural competence requires both SigH and the SigH-dependent operons *comG* and *comE*, which suggests this is an active physiological process and not a physical artefact due to phage tail fragments. Since strains COL and N315 each have resident prophages (φL54a and φN315, respectively), we wished to definitively rule out any possible contribution of phage-mediated “pseudo-transformation". We therefore eliminated the φN315 prophage from N315ex to generate N315ex w/oφ as described in Materials and Methods. As shown in Table 1 (last two columns), strain N315ex w/oφ carrying pRIT-sigH was transformable with pHY300 plasmid DNA isolated directly from an *E*. *coli dam* ^−^ *dcm* ^−^ strain (see Materials and Methods), while the vector control strain, N315ex w/oφ pRIT5H, gave no detectable transformants. This experimental system does not contain any phage particles or phage genes, indicating that SigH-dependent natural competence is phage-independent.

We also demonstrated that unlike natural genetic competence, phage-dependent pseudo-transformation does not require the competence machinery. Indeed, in strain RN4220, which has no resident prophages, we did not obtain any transformants using chromosomal DNA (See Supplementary Material Figure S7). In contrast, when we used RN4220 lysogenized with phage φ11 and the CaCl_2_ washing procedure, we obtained transformants at a very low frequency (between 10^−8^∼10^−9^) through phage-mediated “pseudo-transformation" (See Supplementary Material Figure S7). In this same strain, when we deleted either the *comG* or *comE* genes (strains RKCG and RKCE, respectively; See Materials and Methods and Table 2), there was no significant difference in the number of transformants obtained (See Supplementary Material Figure S7). This indicates that the *comG* and *comE* operons are not required for phage-mediated “pseudo-transformation", in stark contrast to their essential role in SigH-dependent natural genetic competence as shown in Table 1.

**Table 2:** *Staphylococcus aureus* strains and plasmids used in this study.

Strain or plasmid	Description	Source
**Strains**		
COL	MRSA, carrying tetracycline resistance plasmid pT181	[86]
COLw/oφ	COL strain cured of the φL54a prophage	This study
N315	pre-MRSA, Km^R^, Erm^R^	[87]
N315h	N315 carrying pRIT-sigH	[18]
N315comGFP	N315 carrying pRIT-com-gfp	This study
N315aspGFP	N315 carrying pRIT-asp-gfp	This study
N315-GFP	N315 carrying pRIT-gfp	This study
N315ex	SCCmec cured derivative of N315, Km^S^	[88]
N315ex-dr	N315ex pTet-rep pMK3-com-gfp	This study
N315ex-sigH*	*sigH* translation initiation site mutant of N315ex	This study
N315ex-h	N315ex pRIT-sigH	This study
N315ex w/oφ	N315ex cured of the φN315 prophage	This study
N315ex w/oφ h	N315ex w/oφ carrying pRIT-sigH	This study
N315ex w/oφ v	N315ex w/oφ carrying pRIT5H	This study
NKSH	*sigH* disruptant of N315	This study
NKSHh	NKSH carrying pRIT-sigH	This study
N315ΔcomE	N315 Δ*comE* mutant	This study
N315ΔcomEh	N315 ΔcomE pRIT-sigH	This study
N315ΔcomG	N315 Δ*comG* mutant	This study
N315ΔcomGh	N315 ΔcomG pRIT-sigH	This study
8325-4	nonlysogenic derivative of strain 8325N	[89]
RN4220	derivative of 8325-4, restriction minus, modification plus	[90]
RH	RN4220 carrying pRIT-sigH	This study
RNtet-rep	RN4220 carrying pTet-rep	This study
RKCG	RN4220 Δ*comG* mutant, Cm^R^	This study
RKCE	RN4220 Δ*comE* mutant, Cm^R^	This study
A, B, C	SigH active mutants derived from RNtet-rep	This study
A2, B1, C1	plasmid cured strains from A, B, and C	This study
A2b, B1b, C1b	A2, B1, C1 carrying pRITPcomEbga	This study
A2-r, B1-r, C1-r	SigH inactive revertant from A2b, B1b and C1b	This study
MRSA408	MRSA, clinical isolate in Japan	[80]
**Plasmids**		
pHY300PLK	shuttle vector, ori-pAMα1, Amp^R^ (*E. coli*), Tet^R^ (*S. aureus*)	Takara, Japan
pT7gfp	source of *gfp* gene, Amp^R^ (*E. coli*)	[81]
pMK3	shuttle vector, ori-pE194, Amp^R^ (*E. coli*), Km^R^ (*S. aureus*)	[82]
pMK3-com-gfp	P*comG-gfp* transcriptional fusion in pMK3	This study
pRIT5H	shuttle vector, ori-pC194, Amp^R^ (*E. coli*), Cm^R^ (*S. aureus*): used to make pRIT derivatives	[80]
pRIT-sigH	*sigH* overexpressing plasmid, P*spa*, SD sequence modified into SigA type sequence	[18]
pRIT-sigHNH7	*sigH* overexpressing plasmid, P*spa*	This study
pRIT-sigHIRd	*sigH* overexpressing plasmid, half of IR deleted, P*spa*	This study
pRIT-com-gfp	P*comG-gfp* transcriptional fusion	This study
pRIT-asp-gfp	P*asp23-gfp* transcriptional fusion	This study
pRIT-gfp	promoter-less *gfp*	This study
pRIT-PcomE-bgaB	P*comE-bgaB* transcriptional fusion	This study
pTet-rep	P*comG-tet*, P*comE-bgaB*: see Figure 1A	This study
pMAD	ori-pE194^TS^, Amp^R^ (*E. coli*), Erm^R^ (*S. aureus*), P*clpB-bgaB*	[79]
pMAD-tet	pMAD derivative, Amp^R^ (*E. coli*), Erm^R^, Tet^R^(*S. aureus*)	This study
pMADtetcomGII	vector for deletion of *comG* locus, Amp^R^ (*E. coli*), Erm^R^, Tet^R^(*S. aureus*)	This study
pMADtetcomEII	vector for deletion of *comE* locus,Amp^R^ (*E. coli*), Erm^R^, Tet^R^(*S. aureus*)	This study
pMADtet-att	vector for eliminating φN315,Amp^R^ (*E. coli*), Erm^R^, Tet^R^(*S. aureus*)	This study
pMADt492	vector for replacement of *sigH* translation initiation site	This study
pKILts	ori-pYT3^TS^, Amp^R^ (*E. coli*), Tet^R^ (*S. aureus*)	[83]
pKILts-cat	pKILts derivative, Amp^R^ (*E. coli*), Tet^R^, Cm^R^ (*S. aureus*)	[84]

Cm: chloramphenicol, Km: kanamycin, Tet: tetracycline, Erm: Erythromycin.

Taken together, our results have allowed us to demonstrate for the first time that natural genetic competence develops in a SigH-dependent manner in *Staphylococcus aureus*, allowing transformation by extracellular plasmid or chromosomal DNA as well as HGT between different strains.

## Discussion

Since F. Griffith's pioneering discovery of DNA-mediated transformation in *Streptococcus pneumoniae* [41], natural genetic competence in low GC % Gram-positive bacteria has been extensively studied in *Bacillus subtilis* and *S*. *pneumoniae* and shown to involve the assembly of a complex DNA-binding and uptake machinery, made up of a competence pseudopilus and a DNA translocase [13], [16]. During the 1970's, although several reports described “pseudo-transformation" of *S. aureus*, this was revealed to be due in fact to contaminating phage tail fragments mediating DNA entry and HGT [15]. Despite many subsequent attempts, natural genetic competence was never successfully demonstrated in *S*. *aureus* even though sequence analysis readily reveals that its genome carries a practically full repertoire of the required competence gene orthologues, suggesting that specific conditions must exist allowing natural transformation by DNA in *S*. *aureus*. These genes include those encoding the pseudopilus proteins (major pseudopilin ComGC, minor pseudopilins ComGD and ComGE, prepilin peptidase ComC, the ComGB membrane protein and ComGA NTPase) as well as the DNA translocase apparatus (ComEA membrane DNA receptor, ComEC cytoplasmic channel and ComFA ATP-binding protein), displaying amino acid sequence identities ranging from 20 to 70% with the corresponding proteins of *S. pneumoniae* or *B*. *subtilis* (See Supplementary Material Figure S1).

Strong similarities and interesting differences exist between the competence pathways of *B*. *subtilis* and *S*. *pneumoniae*. Indeed, although in both cases the initial triggering event involves a peptide quorum-sensing two-component signal transduction pathway controlling expression of competence genes encoding the DNA uptake machinery, the steps in between are quite different [16], [42], [43]. In *Streptococcus* species, competence genes are regulated by ComX (aka SigX) [30], a secondary sigma factor related to staphylococcal SigH [18] and encoded by duplicated genes (*comX1* and *comX2*) whose expression is directly controlled by the ComDE two-component system [30]. Interestingly, in *B*. *subtilis*, late competence genes are transcribed by the vegetative form of RNA polymerase holoenzyme, Eσ^A^, and instead positively controlled by a specific transcription activator, ComK [44]. Other similarities between the two bacteria include the fact that many additional factors play a part in the production of active ComK or ComX, both of which involve two-component signal transduction networks [42], [43], [45] and the post-transcriptional control of the levels of these two regulatory proteins by the Clp ATP-dependent protease [43], [45]–[51].

In *S*. *aureus*, the situation appears to be more closely related to that of *S*. *pneumoniae*. Indeed, although a protein bearing some similarities to ComK is present (SA0882), we have previously identified the SigH secondary sigma factor, analogous to ComX, and shown that it acts specifically to direct transcription of the *comE* and *comG* operons that encode orthologues of the DNA uptake machinery [18].

Whereas in *S*. *pneumoniae* all of the cells become competent for a short period in time, in *B*. *subtilis* only a maximum of 10% of the cell population achieves competence [43], a fact that has been attributed to the positive autoregulatory feedback loop controlling *comK* expression, generating a heterogeneous bistable response in the cell population [52]–[54]. However, it is important to recall that natural undomesticated strains of *Bacillus subtilis* are in fact considered to be non-competent [55], and that transformation of *B. subtilis* at levels of 10% could be demonstrated only for a few strains isolated following extensive UV and X-ray mutagenesis [56], [57], with the highly transformable 168 strain then chosen for most studies [58]. *B*. *subtilis* strains derived from 168 rapidly became “domesticated" once exposed to the accelerated lifestyle imposed in the laboratory, accumulating multiple mutations affecting competence development and biofilm formation [59]–[61].

Thus, the situation for *Staphylococcus aureus* appears highly reminiscent of that of undomesticated *B*. *subtilis*, with a cryptic DNA uptake apparatus presumably allowing only a very low level of natural transformation in its natural habitat, with the possibility that specific conditions may be required for competence development. Limiting the number of competent recipient cells in a population would be important to sustain genome integrity, minimizing risks and maximizing evolutionary gain by allowing only a fraction of the cells to access genetic variability. Among the many barriers to uptake of foreign DNA, restriction-modification systems are known to play an important role [62]–[64], and tight control of competence gene expression is also required to limit potentially detrimental HGT with other species [43]. In this respect it is tempting to speculate, given the observed population heterogeneity with respect to SigH activity, that natural competence in *S*. *aureus* has evolved as a bet hedging strategy [65], with most of the cells protected against the dangers of HGT, while a fraction are able to increase genetic variability through natural genetic competence.

As ComK and SigX (ComX) are the end products of the regulatory cascades controlling competence development in *B*. *subtilis* and *S*. *pneumoniae*, several attempts have been made to overproduce these proteins in non-competent bacteria in order to obtain genetic transformation. In *Streptococcus pyogenes*, which is not known to become competent, SigX has been shown to control expression of *femB* and *cinA* [50], as well as competence gene orthologues [66]. Likewise, overproduction of SigX in *Lactococcus lactis* also led to increased expression of competence gene orthologues [20]. The recent discovery among *Streptococcus* species of a second quorum-sensing pathway allowing activation of *sigX* expression [67]–[69] has led to the suggestion that members of the pyogenic streptococci group may in fact be able to develop competence under specific conditions [68], [70].

In a similar approach, overproduction of the ComK transcription activator from *B*. *subtilis* was used to induce competence in otherwise non-competent bacteria. This approach was successful both in undomesticated strains of *B*. *subtilis* [55] as well as in *Bacillus cereus*, previously considered to be non-competent [71], [72]. Interestingly, *B*. *cereus* carries two copies of the *comK* gene, reminiscent of the *comX* situation in *S*. *pneumoniae*, although ComK1 and ComK2 appear to play different roles [73]. A recent report reveals that reconstitution of an intact *comK* gene in *Listeria monocytogenes*, leads to expression of competence gene orthologues which play a role in phagosomal escape and virulence, although genetic competence was not tested [74].

**Figure 7:**
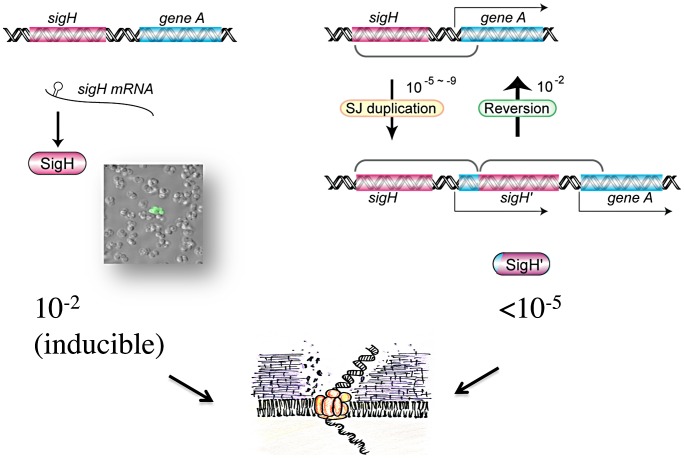
Competence development in *Staphylococcus aureus* involves two distinct mechanisms. A rare SJ-duplication mechanism generates a new chimeric *sigH* gene, and SigH is produced as a fusion protein. The duplication is cured at a high frequency, concomitant with the loss of gene duplication. Under the specific culture conditions used, SigH was expressed stochastically at a frequency of ca. 10^−2^, through a post-transcriptional regulatory mechanism. The inverted repeat sequence upstream of the translation initiation site prevents *sigH* expression, likely forming a secondary structure trapping the ribosome binding site, and/or serving as a post-transcriptional regulatory target, restricting SigH activation to a minor fraction of the cell population. SigH active cells express genes for DNA-binding and uptake machinery and become competent for DNA transformation.

In this study, we have successfully shown for the first time that *S*. *aureus* can develop natural genetic competence for transformation by plasmid or chromosomal DNA, albeit in a minor fraction of the cell population, and that this requires both SigH and the competence genes it controls. In addition to stochastic activation of SigH production, which occurs under specific nutritional conditions during growth in chemically defined CS2 medium, we also showed that expression of *sigH* is controlled post-transcriptionally and that this process involves an inverted repeat sequence that likely acts to sequester the *sigH* ribosome binding site (Figure 7). We cannot exclude that this post-transcriptional control may involve a small regulatory non-coding RNA molecule. Using a positive selection screen, we could show that short junction (SJ) tandem duplication [40] occurred through a chromosomal rearrangement, duplicating the *sigH* gene as a translational fusion with either of the downstream *nusG* or *rplK* genes and effectively relieving expression of the chimeric gene from the post-transcriptional control of the *sigH* 5′ UTR IR element (Figure 7). This process does not appear to be RecA-dependent, since we saw no significant difference in SJ duplication frequencies when cells were treated with Mitomycin C, an efficient inducer of the SOS response (data not shown).

SJ-duplication alters DNA information but is generally unstable and easily cured at higher frequencies than the duplication event, as we show here. To the best of our knowledge, this type of SJ-duplication in the *sigH* locus is the first example in bacteria that generates an in-frame chimeric fusion for gene activation. It remains to be determined whether this type of SJ duplication can occur at any place in the genome in addition to the *sigH* locus. We suggest that the *sigH* gene may be considered as a type of “contingency" locus [75], with a higher mutation rate allowing adaptation to specific deleterious conditions by favoring HGT through DNA uptake. This could explain the mechanism generating what we have termed the “similarity gulf": although the *sigH* gene itself is poorly conserved between different bacterial species, it is nevertheless embedded within a conserved surrounding genetic structure across several bacteria [28].

In *S*. *aureus*, *sigH* has also been reported to direct transcription of phage integrase genes to stabilize the lysogenic state [76]. In the present study, we have shown that SigH is required for competence development in a minor fraction of the cell population. SigH may also protect the subpopulation from phage-induced lytic death, allowing survivors to utilize dead cell materials, including DNA, with a higher probability of acquiring new genes through HGT.

Our demonstration that natural genetic transformation of *S*. *aureus* cells occurs in a SigH-dependent manner helps provide an explanation for the notorious acquisition of antibiotic resistance genes by this major pathogen. Indeed, even at very low natural competence levels, the selective pressures would ensure the survival and rapid spread of strains acquiring antibiotic resistance genes, as currently observed for MRSA strains [7].

Importantly, we showed that a large chromosomal region conferring methicillin resistance (SCC*mec* typeII) could be transferred by transformation. Although it has been suggested that SCC elements less than 45 kb in size may be transferred by phage transduction, no transfer mechanism is known for larger elements such as SCC*mec* typeII. However, it should be noted that we used N315ex cell as the recipient, which had lost the SCC*mec* element, resulting in a MSSA phenotype. The transfer of SCC*mec* into naive MSSA strains needs to be tested, but will require optimization of the transformation protocol. Further efforts will be aimed at improving transformation frequencies in *S*. *aureus* reference strains and understanding the molecular mechanisms involved in the transcriptional and post-transcriptional constraints that have evolved to limit SigH activity to a minor fraction of the cell population. The analysis of the IR sequence in the *sigH* mRNA will also likely play a key role in unraveling the molecular switch involved in controlling competence development.

## Materials and Methods

### Bacterial strains and culture conditions

*S. aureus* strains and plasmids used in this study are listed in Table 2. *S. aureus* was grown in Brain Heart Infusion (BHI) medium, Trypticase Soy Broth (TSB), Nutrient Broth No. 2 (OXOID) supplemented with 3.6 mM CaCl_2_ (NBCaCl_2_), RPMI 1640, or complete synthetic media (CS1, -GS, and CS2). CS1 medium was based on a previous report [77]. The -GS medium was same as CS1, omitting glycine and serine. CS2 synthetic medium was the same as HHWm medium [78] with the following modifications: 30 mg/l guanine, 15 mg/l adenine hemi-sulfate, 8.9 mg/l CaCl_2_, 0.08 mg/l CuSO_4_, 0.17 mg/l ZnSO_4_, 0.12 mg/l CoCl_2_·6H_2_O, 0.12 mg/l Na_2_MoO_4_·2H_2_O (See Supplementary Material Table S1 for full composition).

When using synthetic media, cells were collected from overnight cultures by brief centrifugation, and washed with the appropriate medium to be inoculated. For static anaerobic growth, a 2-ml Eppendorf tube was filled with the medium, and capped to prevent air exchange. For anaerobic growth with shaking, a 100-ml flask containing 50 ml medium was placed in a polyethylene bag together with an AnaeroPack (Mitsubishi Gas Chemical).

### Construction of a positive selection dual reporter plasmid

Oligonucleotides used in this study are listed in Table 3. The *comE* promoter region was amplified with oligonucleotides comEpMlu and comEpKpn2, digested with *Mlu*I and *Kpn*I, and cloned between the corresponding restriction sites of the pMAD shuttle vector [79], replacing the *clpB* promoter with the *comE* promoter. The resultant P*comE-bgaB* transcriptional fusion cassette was excised by *Bam*HI and *Stu*I digestion, and cloned into the *Pvu*II site of pRIT5H [80] by blunt ligation to generate the pRIT-PcomE-bgaB plasmid. The *tet* gene was amplified from pHY300PLK (Takara) with oligonucleotides tetF(Eco) and tetR(Pst), digested by *Eco*RI and *Pst*I, and ligated between the *Eco*RI - *Pst*I sites of pRIT-PcomE-bgaB (See Supplementary Material Figure S8 for plasmid maps of pMAD-PcomE and pRIT-PcomE-bgaB). The resulting plasmid was then treated with *Nde*I and *Eco*RI, and ligated with the *comG* promoter fragment (P*comG*), amplified with PcomG(Sal)p and PcomG(Eco)R, and digested with *Nde*I - *Eco*RI. The ligation mixture was used directly to transform strain RN4220 by electroporation to generate strain RNtet-rep. The structure of the resultant tet-reporter plasmid (pTet-rep) is illustrated in Figure 1A and confers resistance to 12.5 µg/ml chloramphenicol.

**Table 3:** Oligonucleotides used in this study.

Name	Nucleotide sequence
1765R	TTTCGTGCCAGCACCAACCCAACCTTTT
1765F	CCTTGGTTGTATGTCGAAAGAGGGTTTGAA
1792ssbR	TTCAATCGGACCATTTGCATTCGCAAAC
1792ssbF	TATTAGTTGGTCGTTTAACTAGAGACCCAG
3.0-R	CTCAGACAGCAATTTCCCG
492uFSmaI	GCATCCCCCGGGGGAAGGTTTATCGACAG
492uRBglII	AAAGATCTTCTTAGTATTAAACTAACCCC
492dFBglII	GAAGATCTTTAAACAACTCAAGACAG
492dRBglII	AAGAGATCTTCTTCTGGTAACAATGG
agr-up-f	TATGAGGATCCAAATTTATCAATTACCGA
agr-up-r	TTAAGGGATCCCAACTTAATAACCATGTA
agr-D-F(ecoR1)	GGCGAATTCAATTGTAAATCTTGTTGG
agr-down.r(bgl2)	TCAGATCTTTACGAAGCAAATTGGTGGC
ccrA-F	ACGTCAAAGTACGATGAAACAAC
ccrA-R	CTGACTTGTTCTCCAATGTTATCTG
comEpMlu	CTACGCGTGTTTATCATGATATGACATT
comEpKpn2	GAGGTACCAAATCTTTATAGCGTAAT
comG1(Sal)	CGCGTCGACGTTGATACTAATACG
comG2(Bgl)	GACAGATCTCGGCGATAGAGTCTT
down-att	CTAGATCTATTGGTCTGGTGAAAACCATGT
ErmA1	CACGAATATCAGTAAACAAGACAAC
ErmA2	TGCTTCAAAGCCTGTCGGAATTGGT
gfp-F(BamHI)	CGGGATCCGGGAGGCCGTTTCATGAGTAAAGGAGAAGA
gfp-R(PstI)	AACTGCAGCTATTTGTATAGTTCATCCATGCCATGTG
H1p	GGGATCCGATAAATAAGATATTGATT
H2p	TATGTCGACATTCAAAATTTCAAGATG
H5p	GATAATTTGATTAATGAATATAGAGTTACG
IFcomG1	AAAGCTTGGATCCTCTATAGAAATTACAT
IFcomG2	GAGGATCCAAGCTTTTTCGCTTATTGAAATGT
lena007	GGACCAATAATAATGACTAGAGAAG
lena008	CTGAAGGAAGATCTGATTGCTTAAC
mecAF	GTAGTTGTCGGGTTTGGT
mecAR	GGTATCATCTTGTACCCA
NKcomG-U1(Bam)	TAGGATCCGTTGATACTAATACG
NKcomG-U2(Bam)	CAGGATCCTCTATAGAAATTACA
NKcomG-DF(Hind)	GTCAAAGCTTTTTCGCTTATTGA
NKcomG-D(Hind)	TTGAAGCTTCGGCGATAGAGT
NKcomE-UF(Bam)	ATGGATCCAAGTGTTGATATGTTG
NKcomE-UR2(Bam)	ACTGGATCCTTAGATGTCATTTTA
NKcomE-DF(Hind)	ATTAAGCTTATGGATGTAAGACA
NKcomE-DR(Hind)	AAAAGCTTCGGTAATTACTTGTA
PcomG(Eco)R	GTGAATTCCTCGCTTTCATTTCTATCGCT
PcomG(Sal)p	GAAGGTCGACATATGTGTTCGATGAATTCG
SA0492Rp	TCGTTGTATGGCATTATAAATCACTTTCTC
SA1374up-F(NdeI)	TAACATATGTGTTCGATGAATTCGCAGTTGTTGG
SA1374up-R(BamHI)	CGGGATCCCTCGCTTTCATTTCTATCGCT
SA1986up-f(NdeI)	TTCCATATGTTAATTAAACATCGTGACCCAG
SA1986up-r(Bam)	CGGGATCCAGGATAAAGTTTTTAAGTCGT
sigH-D(Sal)	CCTAAGTCGACTTTCAAATCATTTTG
sigH-f(Eco)	CGAATTCTTGAAATTTTGAATGTAAC
sigH-F(-)	GTCTAGAAGATAGAAGGGGTTAGT
sigHf2(Eco)	AAGAATTCTTAAATTTTAGATAGAAGG
NKSH-F(BamHI)	CGGGATCCATTAATTGACAACTCAAGACA
NKSH-R(HindIII)	CCCAAGCTTTTATTGTTCACACATTAAATA
tetF(Eco)	CTGAATTCATTATTGGAGGGTGAAA
TetF(Sal)	CATATTGTCGACTAAGTGATGAAATACTG
TetR(EcoRI)	GGAATTCCTGTTATAAAAAAAGGATCAAT
TetR(Xho)	GAACTCGAGTTATAAAAAAAGGATCA
tetR(Pst)	AACTGCAGTTATAAAAAAAGGATCA
up-att	CGAATTCGGAACTTGATAGTTTCTTTTAGC
Xsau325	GGATCAAACGGCCTGCACA

### Isolation of SigH active cells and revertants

RNtet-rep and other reporter strains were grown in BHI containing 12.5 µg/ml chloramphenicol at 37°C overnight. 100 µl aliquots were inoculated into 100 ml drug-free TSB and grown at 37°C with shaking until the stationary phase. About 2×10^9^ cells and tetracycline (5 µg/ml) were mixed into TSB-agar (TSA) at 50°C. The mixture was immediately poured into sterile plates and solidified at room temperature. The plates were incubated at 37°C for 2 days. Colonies were replicated on TSA plates containing 5 µg/ml tetracycline and 100 µg/ml X-gal. Percentages of blue colonies were determined to calculate the SigH activation frequency.

Three SigH active clones generated from RNtet-rep were selected for further analysis. To eliminate the reporter plasmid, they were submitted to a few cycles of growth at 42°C and 37°C in drug free TSB medium, and plated onto TSA containing 100 µg/ml X-gal. White colonies with the expected phenotypes (tet^S^, cm^S^) were selected as plasmid cured strains, and designated A2, B1, and C1. pRIT-PcomE-bgaB was introduced into by electroporation into these strains to confirm stable SigH activation, giving strains A2b, B1b, and C1b. In order to isolate revertants of SigH activity, strains A2b, B1b, and C1b were grown in drug free BHI medium, and plated on TSA containing 100 µg/ml X-gal and 12.5 µg/ml chloramphenicol. White colonies were tested by Southern blot analysis for the loss of *sigH* duplication.

### Fluctuation test

Strains RNtet-rep and N315 carrying pTet-rep were grown overnight at 37°C in TSB containing 12.5 µg/ml chloramphenicol. A 1.4 µl aliquot was used to inoculate 140 ml of drug-free TSB. 2 ml aliquots were distributed into 15∼20 test tubes and grown overnight at 37°C, while 100 ml was grown in a single 500-ml flask. One ml of the culture was harvested from each test-tube or flask, and mixed with TSA containing 5 µg/ml tetracycline at 50°C. Numbers of SigH active cells (tet^R^, β-galactosidase positive) were then determined as described above.

### Southern hybridization analysis

*S*. *aureus* genomic DNA was purified using standard procedures. One µg of DNA was digested with *Hin*dIII or *Pvu*II and separated by electrophoresis on a 1% agarose gel. The separated DNA fragments were transferred to a Hybond-N+ membrane (Amersham Biosciences) by Southern blot. Analysis was carried out using the AlPhos Direct labeling kit and the CDP star detection reagent system according to the manufacturer's instructions (Amersham Biosciences). DNA fragments used to prepare the probes were amplified by PCR with oligonucleotides H1p and H2p for the upstream region, and H5p and SA0492Rp for the *sigH* coding sequence.

### Construction of GFP reporter strains

A 746 bp DNA fragment carrying the *gfp* (green fluorescent protein) gene with its Shine Dalgarno sequence, GGGAGG, was amplified by PCR from the pT7gfp vector [81] using oligonucleotides gfp-F(BamHI) and gfp-R(PstI). Promoter regions were PCR-amplified from N315 using oligonucleotides SA1374up-F(NdeI) and SA1374up-R(BamHI) for *comG* (positions −253 to +65 from the translational start site), or SA1986up-f(NdeI) and SA1986up-r(Bam) for *asp23* (−208 through +65). The promoter region and *gfp* DNA fragments were digested with the appropriate restriction enzymes and cloned together between the *Nde*I-*Pst*I sites of the pRIT5H shuttle vector. The resultant plasmids, pRIT-com-gfp and pRIT-asp-gfp, carry transcriptional fusions between the *comG* or *asp23* promoter regions and the *gfp* gene. These plasmids were introduced into *S*. *aureus* N315 by electroporation to generate strains N315comGFP and N315aspGFP. The negative control plasmid, pRIT-gfp, has no promoter sequence.

To construct pMK3-comGFP, the *comG* promoter and *gfp* region from pRIT-com-gfp was amplified by PCR with oligonucleotides PcomG(Sal)p and gfp-R((PstI), and ligated between the *Sal*I-*Pst*I sites of the pMK3 shuttle vector [82].

### Western blot analysis

Total protein extracts from *S. aureus* cells were prepared as previously described [18], except that cells were suspended in a buffer containing 20 mM Tris-HCl (pH7.5), 5 mM EDTA, 1 mM PMSF, and 0.05 mg/ml lysostaphin. After cell wall degradation, SDS was added at a final concentration of 1%. Fifteen µg of total proteins were separated by SDS-PAGE and transferred onto PVDF membranes. Western blot detection of SigH was previously described [18]. For GFP detection, the membrane was incubated with 2 µg/ml of BD Living Colors Peptide Antibody (BD Biosciences) for 16–20 h at 4°C. After a wash with TBST, the membrane was incubated in the presence of 5 µg/ml of Goat Anti-Rabbit IgG Antibody conjugated with HRP (Promega) at 25°C for 30 min. Immuno-reactive bands were detected using the ECL plus Western Blotting Detection System (Amersham Biociences). For Figures 5C and 6B the blot was treated with 10 µg/ml Anti-GFP IgY (Aves Labs) as the first antibody and 0.7 µg/ml of Anti-Chicken IgY HRP Conjugate (Promega) as the second antibody by using the SNAPi.d. Protein Detection System (Millipore), and signals were detected using the Amersham ECL Western Blotting Detection System (GE Healthcare).

### Confocal microscopy

Confocal imaging of cells was performed using a confocal laser-scanning microscope (TCS-SP2, Leica microsystems). GFP was excited at 488 nm using the blue laser and fluorescence images were collected using the green channel. All fluorescence images were obtained with the same settings. Serial optical sections were obtained at about 0.6 µm intervals and three of them per image were reconstituted using the LCS Lite software (Leica Microsystems).

### Construction of the NKSH *sigH* inactivation mutant

The *sigH* gene was inactivated by insertional mutagenesis in strain N315 using the method previously described [83]. The *sigH* insertional vector was constructed using the pKILts plasmid and a *sigH* DNA fragment (nucleotides -1 to +473) amplified by PCR with oligonucleotides NKSH-F(BamHI) and NKSH-R(HindIII). The correct homologous recombination in the resulting *sigH* inactivation mutant, strain NKSH, was confirmed by PCR and Southern blot analysis. The pRIT-sigH [18] plasmid was introduced into NKSH *sigH* mutant to generate the complemented strain NKSHh.

### Construction of *sigH* expression plasmids

Construction of pRIT-sigH was previously described [18]. The pRIT-sigHNH7 plasmid was constructed by inserting a PCR fragment amplified using oligonucleotides sigH-f(Eco) and sigH-D(Sal) between the *Eco*RI-*Sal*I sites of pRIT5H. For pRIT-sigHIRd construction, primers sigHf2(Eco) and sigH-D(Sal) were used.

### Northern hybridization analysis

Cells were harvested by centrifugation at 5,000× *g* at 4°C. Total RNA was extracted as previously described [83]. For Figure 6D, total RNA was treated with TurboDNA-free (Ambion) to eliminate any trace of the vector DNA. Fifteen µg (for Figure 3C) or 5 µg (for Figure 6D) of total RNA was separated on a 1% agarose-formamide denaturing gel and transferred onto a Hybond N+ membrane (Amersham Biosciences). In Figure 3C, DNA fragments prepared by PCR from the N315 genomic DNA were labeled with [α^32^P]-dCTP by random priming using the Ready-to-Go DNA labeling Beads (Amersham Biosciences), and used as probes. Hybridizations were carried out at 60°C in a solution containing 5× SSPE, 5× Denhardt's solution, 0.5% SDS, and 20 µg/ml of salmon sperm DNA, and the final washing condition was at 50°C in 0.1× SSC and 0.1% SDS. In Figure 6D, probe preparation and detection was carried out using AlkPhos Direct Labelling and Detection System with CDP-*Star* (GE Health Care). The *sigH* PCR fragment amplified with sigH-F(−) and SA0492Rp was used as a probe.

### Site-directed mutagenesis of *sigH* translation initiation codons

Potential translation initiation codons (TTG) of *sigH* were changed to Ochre stop codons (TAA) by PCR-based site-directed mutagenesis (illustrated in Figure 5B). Two oligonucleotides, 492uRBglII and 492dFBglII, were designed to include these mutations and a *Bgl*II site. The *tet* gene was amplified with oligonucleotides Tet-F(Sal) and Tet-R(EcoRI) using pHY300PLK (Takara) as a template, and ligated between the *Eco*RI-*Sal*I sites of pMAD to generate pMADtet (See Supplementary Material Figure S8 for plasmid map). Oligonucleotides 492uFSmaI & 492uRBglII were used to amplify the *sigH* upstream region, and 492dFBglII & 492dRBglII for the downstream region. These fragments were sequentially ligated between the *Sma*I-*Bgl*II sites of pMADtet to generate the pMADt492 targeting plasmid. This was introduced into N315ex by electroporation. Mutants (tetracycline sensitive, β-galactosidase negative) were selected as previously described [79], and designated N315ex-sigH*. The introduced mutations were confirmed by direct sequencing analysis.

### Construction of *comG* and *comE* mutants

Deletion/replacement mutants of the *comG* and *comE* regions were constructed by double-crossover homologous recombination. The targeting cassettes were constructed using plasmid pKILts-cat [84]. Oligonucleotide pairs used to amplify DNA fragments encompassing the *comG* (from *SA1374* through *SA1369*) and *comE* regions (from *SA1418* through *SA1416*) were: NKcomG-U1(Bam) and NKcomG-U2(Bam) for the *comG* upstream region; NkcomG-DF(Hind) and NKcomG-D(Hind) for the *comG* downstream region; NKcomE-UF(Bam) and NKcomE-UR2(Bam) for the *comE* upstream region; NKcomE-DF(Hind) and NKcomE-DR(Hind) for the *comE* downstream region (Table 3). The *comG* targeting cassette constructed in pKILts-cat was amplified by PCR with oligonucleotides comG1(Sal) and comG2(Bgl) and cloned between the *Sal*I-*Bgl*II sites of pMAD [79]. The *comG* and *comE* deletion/replacement mutant strains (RKCG and RKCE) were then constructed in strain RN4220 by integration/excision as previously described [79], [84]. The absence of each region was confirmed by PCR and Southern blot analysis.

Markerless *comG* and *comE* deletion mutants of N315 were also constructed using the pMAD vector. The chloramphenicol resistance gene was first eliminated from pMADcomG by inverse PCR using oligonucleotides IFcomG1 and IFcomG2, followed by self-circularization using the In-Fusion system (Clontech). The tetracycline resistance gene was amplified from pHY300PLK using oligonucleotides TetF(Sal) and TetR(Xho), and inserted into the *Sal*I site to generate the *comG* locus targeting vector, pMADtetcomGII. To construct the *comE* targeting vector, the upstream and downstream regions were amplified by PCR using oligonucleotides 2EUF(Eco) and 2EUR(Bgl) (upstream), and 2EDF(Bgl) and 2EDR(Bgl) (downstream). These fragments were sequentially inserted into the *Eco*RI-*Bgl*II sites and the *Bgl*II site of pMAD-tet, respectively, to generate pMADcomEII. Plasmids were introduced into strainN315 by electroporation, after passaging through strain RN4220. Mutants (tetracycline sensitive, β-galactosidase negative) were selected as described above. The N315ΔcomE and N315ΔcomG mutants lack the same region deleted in strains RKCG and RKCE, respectively, but have no antibiotic resistance marker at the deleted locus.

### Natural transformation of *S*. *aureus* cells

The φL54a prophage was cured from the COL strain by ultraviolet light treatment as described [85]. The resulting strain, COLw/oφ, was verified for φL54a susceptibility and restored lipase activity as described. Plasmid pT181 DNA was then purified from strain COLw/oφ, using the QIAfilter Plasmid Midi kit (QIAGEN).

Competent *S. aureus* recipient cells were prepared by overnight growth in TSB containing chloramphenicol (12.5 µg/ml) with shaking at 37°C. Cells were harvested from five hundred µl of overnight culture, washed with CS2 medium, resuspended in 10 ml of CS2 medium and grown at 37°C with shaking. After 8 hours, cells were harvested by centrifugation, and resuspended in 10 ml of fresh CS2 medium. Ten µg of plasmid DNA (pT181 or pHY300 isolated from *E. coli* HST04 *dam*-/*dcm*-) was added to the suspension, and incubation was pursued at 37°C with shaking for 2 hours. Cells were mixed into melted BHI-agar pre-cooled to 55°C together with 5 µg/ml tetracycline and 5 µg/ml erythromycin, and incubated at 37°C for 2 days. Colonies were tested for their characteristics to confirm that they were *bona fide* recipient cell transformants (e.g. kanamycin resistance and plasmid species). For transformation with chromosomal DNA, 10 µg of N315 genomic DNA was added to the cells. Transformants were selected with 100 µg/ml kanamycin (see Supplementary Material Figure S3).

### Strain-to-strain plasmid transformation

Cells were grown overnight in TSB with shaking at 37°C. One hundred µl of donor (COL) and 400 µl of recipient cells (N315 derivative) were mixed and washed with CS2 medium. Cells were resuspended in 10 ml of CS2 medium, and grown at 37°C for 8∼10 hours with shaking. CFU values of N315 derivatives (larger colonies than COL derivatives) after co-cultivation were counted on drug-free BHI-agar plate. Co-cultivated cells were mixed into melted BHI-agar pre-cooled to 55°C together with 5 µg/ml tetracycline and 5 µg/ml erythromycin, and incubated at 37°C for 2 days. Colonies were tested for susceptibility to kanamycin to verify the transformants and kanamycin resistant clones were regarded as N315 derivatives.

### Elimination of φN315 from strain N315ex

The φN315 prophage is integrated at an *att* site located within the *hlb* β-hemolysin gene, inactivating the gene and abolishing beta-hemolysis. We used the pMAD system to precisely excise φN315 from the N315 genome. A set of primers, up-att and down-att, was designed upstream and downstream of *hlb* (See Table 3). The target region encompassed by these primers is 46 kbp when phage φN315 is present, and 2.4 kbp when the phage is excised. We noted that φN315 is spontaneously excised from the genome in a minor fraction of the cell population, and that the intact *hlb* gene can be amplified by PCR (data not shown). The latter fragment was amplified from N315 genomic DNA and cloned between the *Eco*RI and *Bgl*II sites of pMAD-tet, generating pMAD-tet-att. The plasmid was then introduced into strain N315ex and the phage-cured N315ex strain was selected by repeated integration/excision cycles of pMAD-tet-att as previously described [79]. In addition, beta-hemolysin activity was also monitored to verify the successful phenoconversion. The absence of the *SA1765* and *SA1792* (*ssb*) phage genes in the resultant N315ex w/oφ was confirmed by PCR (primers 1765F and 1765R for *SA1765* and 1792ssbR and 1792ssbF for *ssb*) and Southern blot analysis using the 1765F–1765R PCR-generated DNA fragment as a probe.

### Pseudo-Transformation assays

Phage particle-dependent pseudo-transformation assays were carried out based on the CaCl_2_ washing method previously described [77] with some modifications. In brief, *S. aureus* cells were grown in TSB medium at 37°C overnight with shaking (180 rpm, BR-23UM: TAITEC). Cells were recovered by centrifugation and washed once with 0.1 M Tris-malate (pH 7.0). The cells were resuspended in 0.1 M Tris-malate (pH 7.0) supplemented with 0.1 M CaCl_2_. 24 µg of purified N315 genomic DNA was added to 1 ml of the cell suspension, and incubated at room temperature for 40 min. Cells were recovered by centrifugation and suspended in drug-free BHI medium. Following 1 h incubation at 37°C with shaking, cells were mixed with molten BHI-agar medium pre-cooled to 55°C and supplemented with 5 µg/ml erythromycin and poured into plates. After two days incubation at 37°C, colonies were counted and checked for the presence of the *erm* gene by PCR with primers ErmA1 and ErmA2.

## Supporting Information

Figure S1 **Comparison of competence genes and their organization in** ***Staphylococcus aureus*** **,** ***Bacillus subtilis*** **and** ***Streptococcus pneumoniae*** **.**
(TIF)

Figure S2 **Summary of the frequencies of SigH active Tet^R^ BgaB-positive colonies in several independent experiments and different genetic backgrounds.** Reporter strains carrying the positive selection tetracycline resistance (*tet*) reporter plasmid (pTet-rep) were grown in drug-free TSB and then selected with 5 µg/ml tetracycline. Shown is the summary of independent experiments from different cultures, while the data in Figure 1B is from a single overnight culture showing a lower fluctuation.
(TIF)

Figure S3 **Nucleotide sequences of SJ duplication junctions in** ***sigH*** **chimeric gene fusions.**
(TIF)

Figure S4 **Western blot analysis of SigH-dependent GFP production under different growth conditions A**) Western blot analysis of SigH-dependent GFP production in strain N315comGFP. Cells were grown at 37°C in -GS or CS1 medium for 24 or 48 hours with or without shaking under aerobic (upper panel) or anaerobic (lower panel) conditions. Results with the highest induction efficiency are shown, with a tendency indicating that static growth rather than shaking could enhance SigH-dependent GFP production. The removal of glycine and serine from CS1 medium improved SigH activation (-GS medium). **B**) Time course of GFP induction in N315comGFP cells during anaerobic static growth in -GS medium. Cells were grown in separate tubes and harvested at the indicated times, suggesting that GFP induction seems to be a specific cellular response rather than a spontaneous event.
(TIF)

Figure S5 **Plasmid profile verification of** ***S*** **.** ***aureus*** **transformants.** *S*. *aureus* cells were transformed with plasmid pT181, and plasmids were purified from twelve transformants using the GeneElute Plasmid Miniprep Kit (Sigma) and submitted to agarose gel electrophoresis. Strain N315 carries an endogenous 24.6 kb plasmid, pN315. Strain N315h carries the pRITsigH SigH production plasmid in addition to pN315. M: λ *Hin*dIII DNA fragments.
(TIF)

Figure S6 **PCR-based verification of SCC** ***mec*** **II element in** ***S*** **.** ***aureus*** **chromosomal DNA transformants.** **A**) PCR verification of transformants for the horizontal transfer of chromosomal determinants. The SCC*mec*II element of N315 carries the *aadD* gene for kanamycin resistance (within pUB110), as well as the *mecA* gene conferring oxacillin (OX) resistance, located 3.44 kilobase pairs apart. Strain N315ex is an N315 derivative that has lost the complete SCC*mec*II region. N315ex-h cells (N315ex strain carrying the pRIT-sigH plasmid; Km^S^, OX^S^) were transformed with purified N315 genomic DNA. Transformants were first selected for kanamycin resistance (100 µg/ml). The presence of the *aadD* gene was tested by PCR using primers lena007 and lena008 (top panel, typical profiles of selected colonies), Forty-three out of forty-seven kanamycin resistant colonies carried the *aadD* gene (c.a. 90%), with the remaining 4 likely spontaneous mutants. The *aadD* transformation efficiency was 3.1×10^−8^. The presence of the *mecA* gene was verified by PCR using oligonucleotides mecAF and mecAR (bottom panel), and was detected in 37 out of the 43 *aadD*-positive transformants. Positive and negative controls were carried out using chromosomal DNA from the donor and recipient strains (N315 and N315exh, respectively; last two lanes). **B**) Long PCR experiments confirming transfer by transformation of the entire SCC*mec*II element in seven transformants where *aadD* and *mecA* were both present. Primer locations are shown on the SCC*mec*II map. All of the transformants gave signals with the expected size. Chromosomal DNA from strains N315 and N315ex-h was used for positive and negative controls, respectively. M: λ *Hin*dIII, M_2_: 1 kb ladder (NEB).
(TIF)

Figure S7 **Phage-dependent “pseudo-transformation" in** ***S*** **.** ***aureus*** **does not require the** ***comG*** **and** ***comE*** **operons, unlike natural genetic competence.** Experiments were carried out based on the method previously described [77] with some modifications. In brief, *S. aureus* cells were grown in TSB medium at 37°C overnight with shaking (180 rpm). Cells were recovered by centrifugation, washed once with 0.1 M Tris-malate (pH 7.0) and resuspended in 0.1 M Tris-malate (pH 7.0) supplemented with 0.1 M CaCl_2_. 24 µg of the N315 purified genomic DNA was added to 1 ml of cell suspension, and cells were incubated at room temperature for 40 min. Cells were recovered by centrifugation, suspended in drug-free BHI medium and incubated for 1 h at 37°C with shaking (100 rpm). Cells were mixed with BHI-agar medium pre-cooled to 55°C and supplemented with 5 µg/ml erythromycin and poured into plates. After two days incubation at 37°C, colonies were counted and checked for the presence of the *erm* gene by PCR with primers ErmA1 and ErmA2.
(TIF)

Figure S8 **Plasmid maps of plasmids pMAD-PcomE, pRIT-PcomE-bgaB, and pMAD-tet.** Plasmids pMAD-PcomE and pRIT-PcomE-bgaB are intermediates used for constructing plasmid pTet-rep. Plasmid pRIT-PcomE-bgaB was used as a reporter to follow SigH activity in Figure 1C. The Pspa promoter sequence is located between the *Nde*I and *Eco*RI sites, and hence does not affect expression of the P*comE-bgaB* transcriptional fusion. Plasmid pMAD-tet was modified from pMAD for use in *S. aureus* strains resistant to erythromycin.
(TIF)

Table S1 **Composition of CS2 medium.**
(PDF)
