# Supplementary figures and images for: Expression of a Cryptic Secondary Sigma Factor Gene Unveils Natural Competence for DNA Transformation in Staphylococcus aureus
Source: PLoS Pathog. 2012 Nov 1;8(11):e1003003. doi: 10.1371/journal.ppat.1003003 (PMC3486894; doi:10.1371/journal.ppat.1003003)

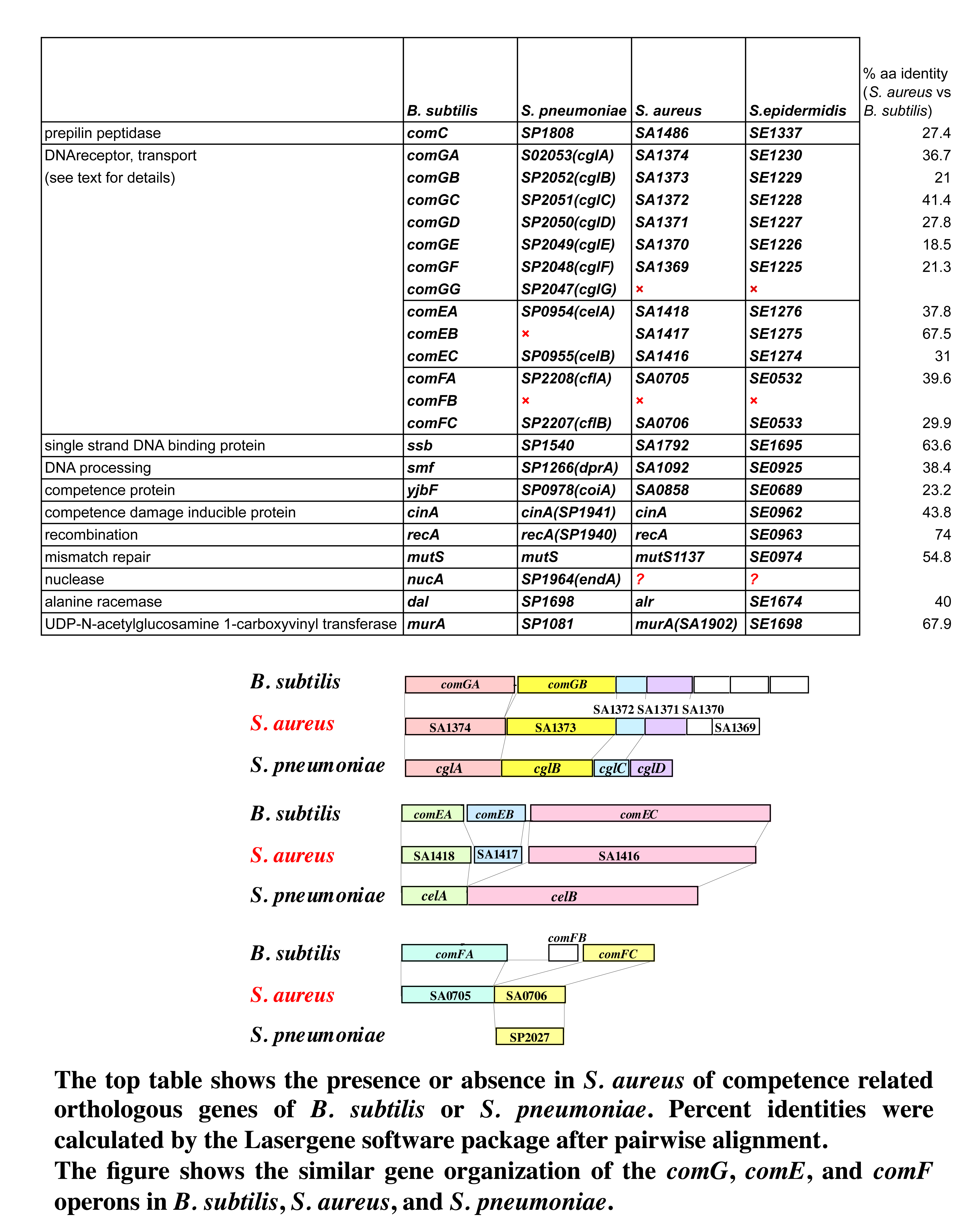

Supplement: Figure S1 — Comparison of competence genes and their organization in Staphylococcus aureus , Bacillus subtilis and Streptococcus pneumoniae . (TIF) [file ppat.1003003.s001.tif]

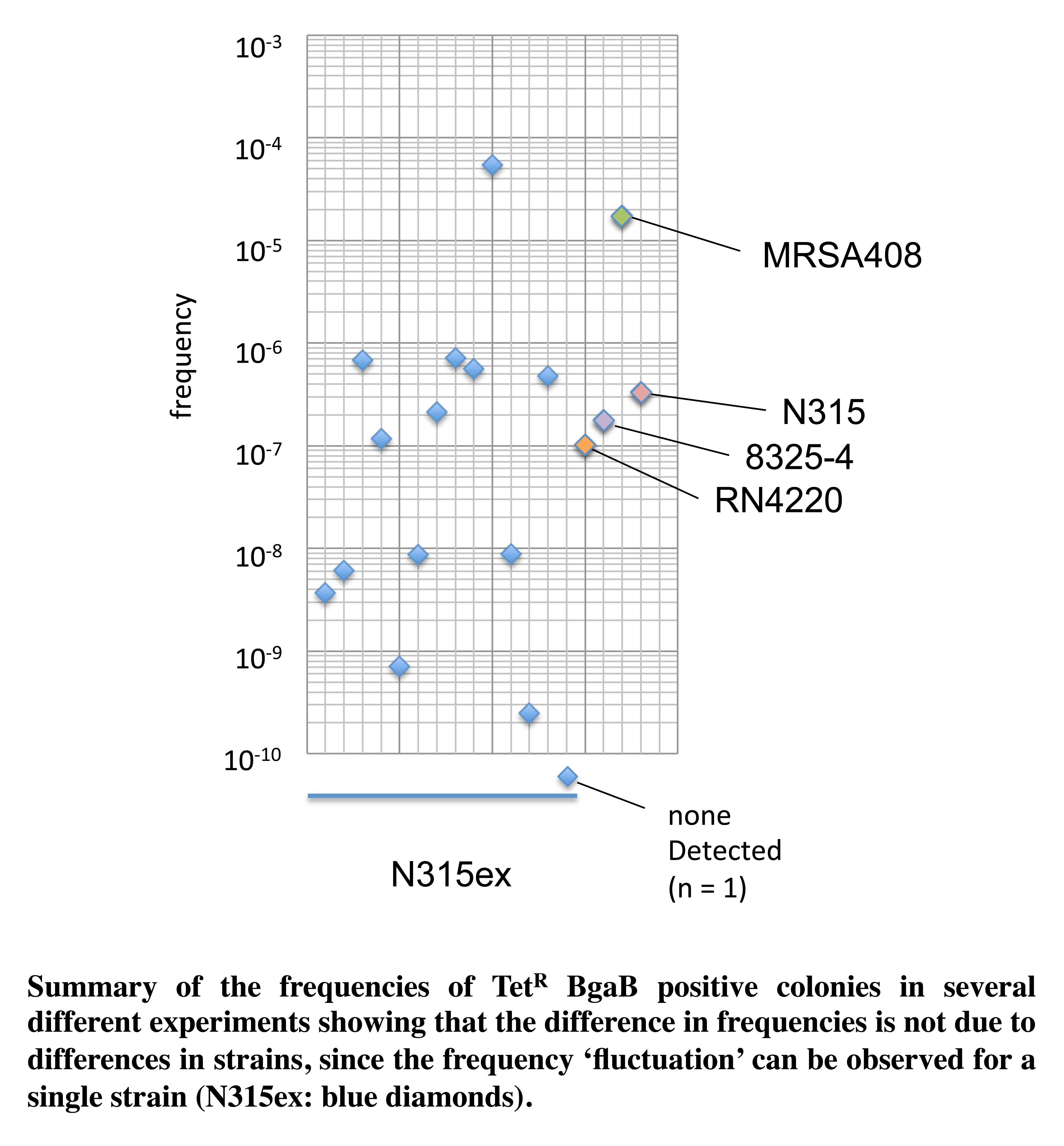

Supplement: Figure S2 — Summary of the frequencies of SigH active TetR BgaB-positive colonies in several independent experiments and different genetic backgrounds. Reporter strains carrying the positive selection tetracycline resistance (tet) reporter plasmid (pTet-rep) were grown in drug-free TSB and then selected with 5 µg/ml tetracycline. Shown is the summary of independent experiments from different cultures, while the data in Figure 1B is from a single overnight culture showing a lower fluctuation. (TIF) [file ppat.1003003.s002.tif]

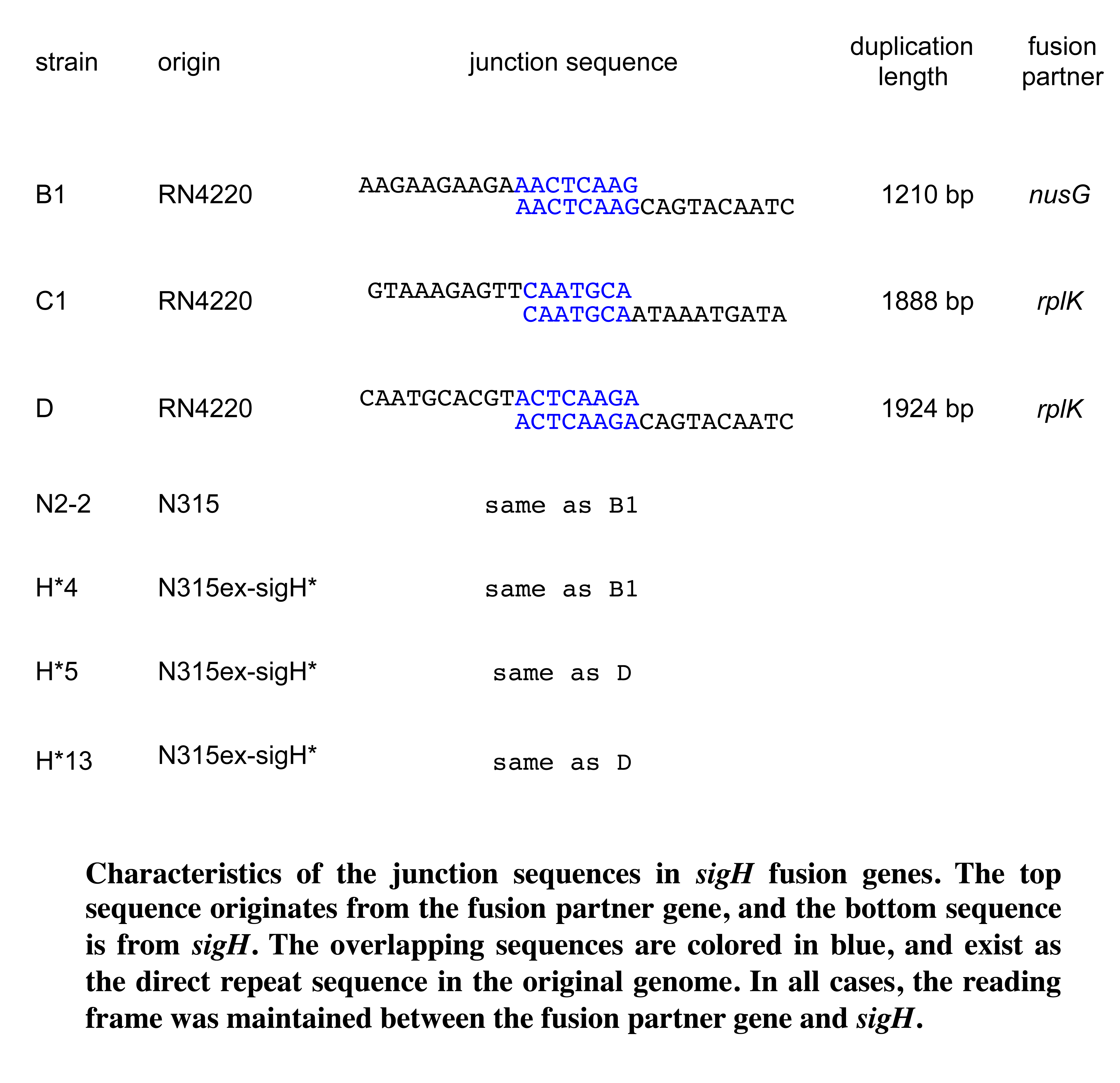

Supplement: Figure S3 — Nucleotide sequences of SJ duplication junctions in sigH chimeric gene fusions. (TIF) [file ppat.1003003.s003.tif]

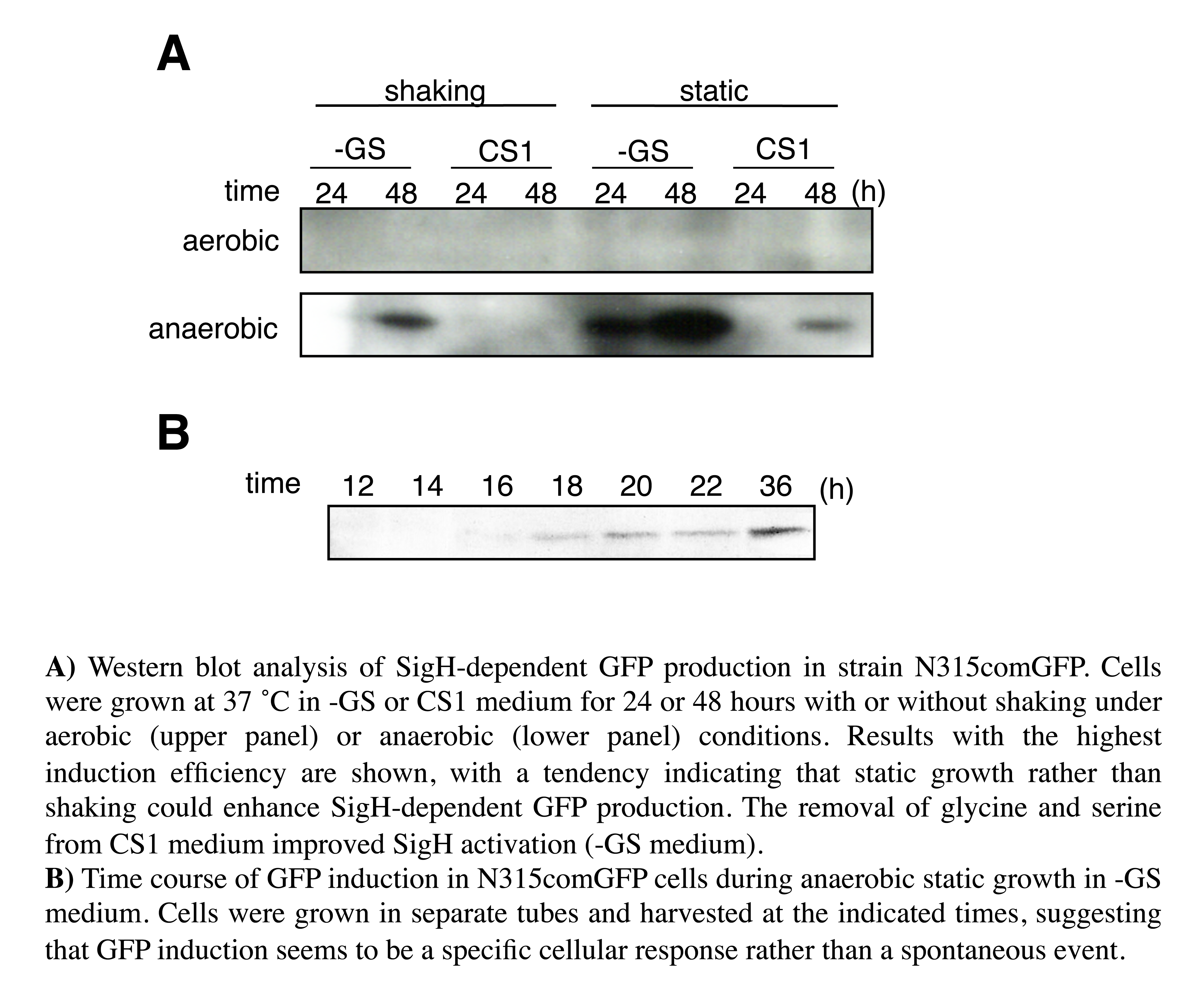

Supplement: Figure S4 — Western blot analysis of SigH-dependent GFP production under different growth conditions A) Western blot analysis of SigH-dependent GFP production in strain N315comGFP. Cells were grown at 37°C in -GS or CS1 medium for 24 or 48 hours with or without shaking under aerobic (upper panel) or anaerobic (lower panel) conditions. Results with the highest induction efficiency are shown, with a tendency indicating that static growth rather than shaking could enhance SigH-dependent GFP production. The removal of glycine and serine from CS1 medium improved SigH activation (-GS medium). B) Time course of GFP induction in N315comGFP cells during anaerobic static growth in -GS medium. Cells were grown in separate tubes and harvested at the indicated times, suggesting that GFP induction seems to be a specific cellular response rather than a spontaneous event. (TIF) [file ppat.1003003.s004.tif]

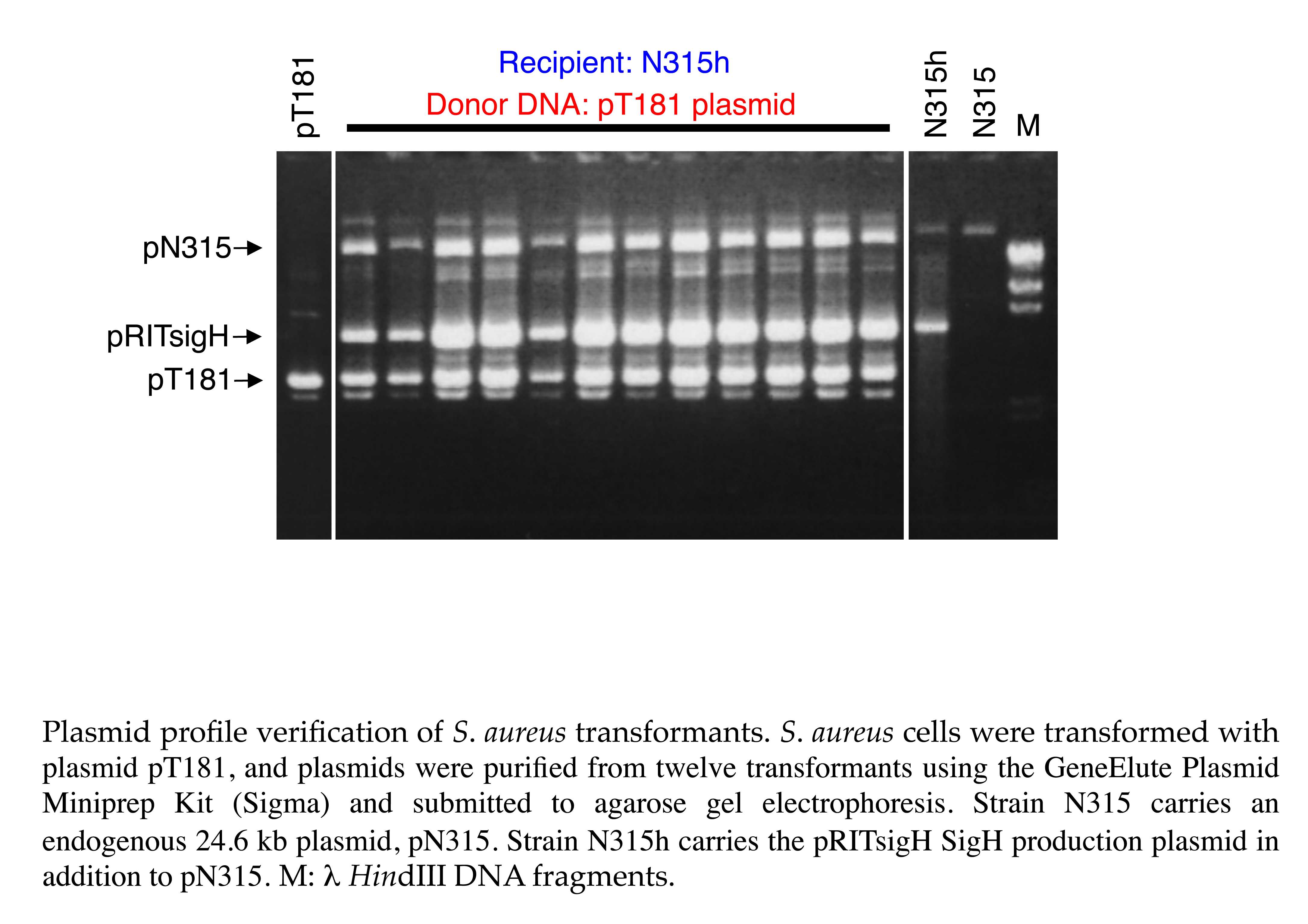

Supplement: Figure S5 — Plasmid profile verification of S . aureus transformants. S. aureus cells were transformed with plasmid pT181, and plasmids were purified from twelve transformants using the GeneElute Plasmid Miniprep Kit (Sigma) and submitted to agarose gel electrophoresis. Strain N315 carries an endogenous 24.6 kb plasmid, pN315. Strain N315h carries the pRITsigH SigH production plasmid in addition to pN315. M: λ HindIII DNA fragments. (TIF) [file ppat.1003003.s005.tif]

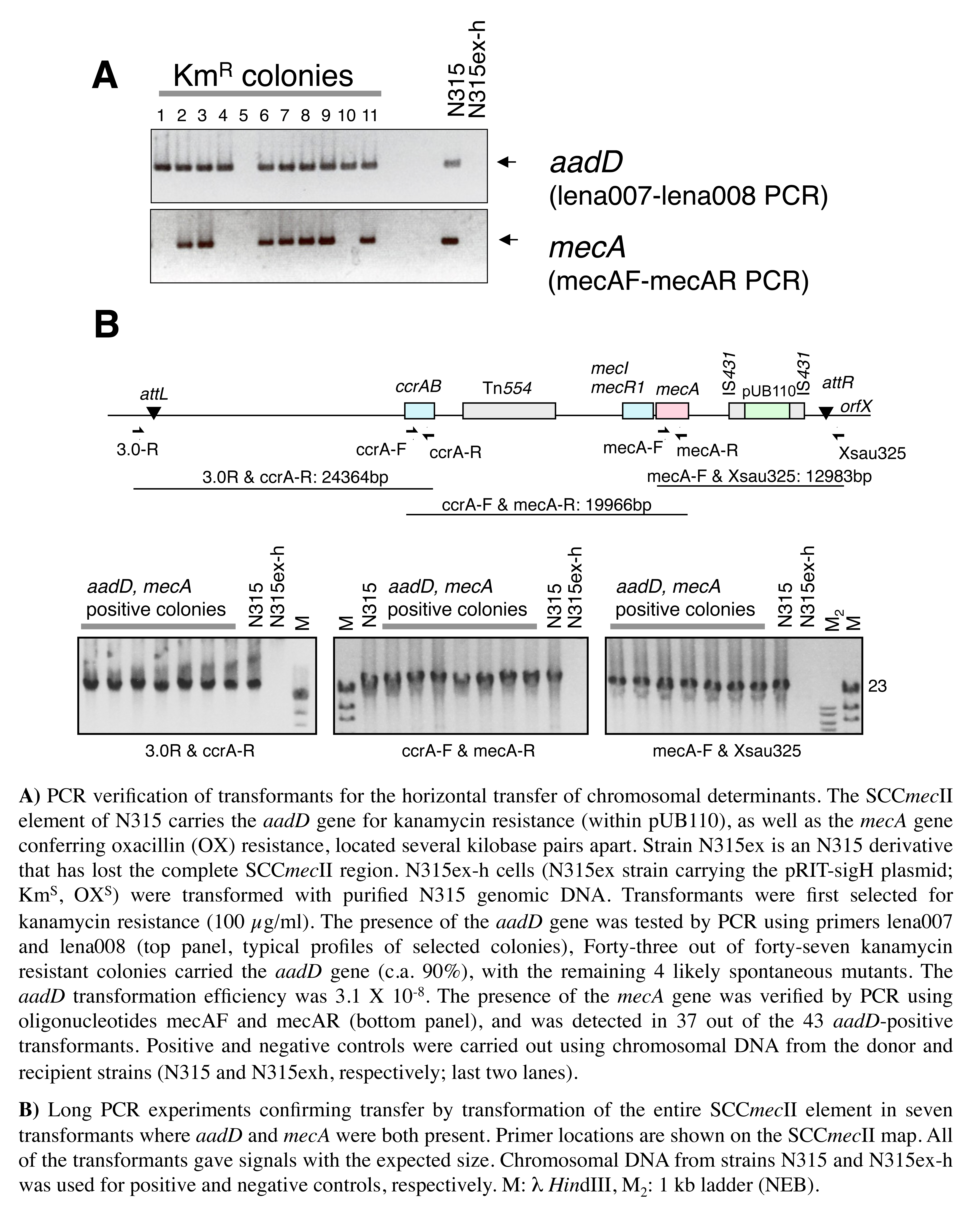

Supplement: Figure S6 — PCR-based verification of SCC mec II element in S . aureus chromosomal DNA transformants. A) PCR verification of transformants for the horizontal transfer of chromosomal determinants. The SCCmecII element of N315 carries the aadD gene for kanamycin resistance (within pUB110), as well as the mecA gene conferring oxacillin (OX) resistance, located 3.44 kilobase pairs apart. Strain N315ex is an N315 derivative that has lost the complete SCCmecII region. N315ex-h cells (N315ex strain carrying the pRIT-sigH plasmid; KmS, OXS) were transformed with purified N315 genomic DNA. Transformants were first selected for kanamycin resistance (100 µg/ml). The presence of the aadD gene was tested by PCR using primers lena007 and lena008 (top panel, typical profiles of selected colonies), Forty-three out of forty-seven kanamycin resistant colonies carried the aadD gene (c.a. 90%), with the remaining 4 likely spontaneous mutants. The aadD transformation efficiency was 3.1×10^−8^. The presence of the mecA gene was verified by PCR using oligonucleotides mecAF and mecAR (bottom panel), and was detected in 37 out of the 43 aadD-positive transformants. Positive and negative controls were carried out using chromosomal DNA from the donor and recipient strains (N315 and N315exh, respectively; last two lanes). B) Long PCR experiments confirming transfer by transformation of the entire SCCmecII element in seven transformants where aadD and mecA were both present. Primer locations are shown on the SCCmecII map. All of the transformants gave signals with the expected size. Chromosomal DNA from strains N315 and N315ex-h was used for positive and negative controls, respectively. M: λ HindIII, M2: 1 kb ladder (NEB). (TIF) [file ppat.1003003.s006.tif]

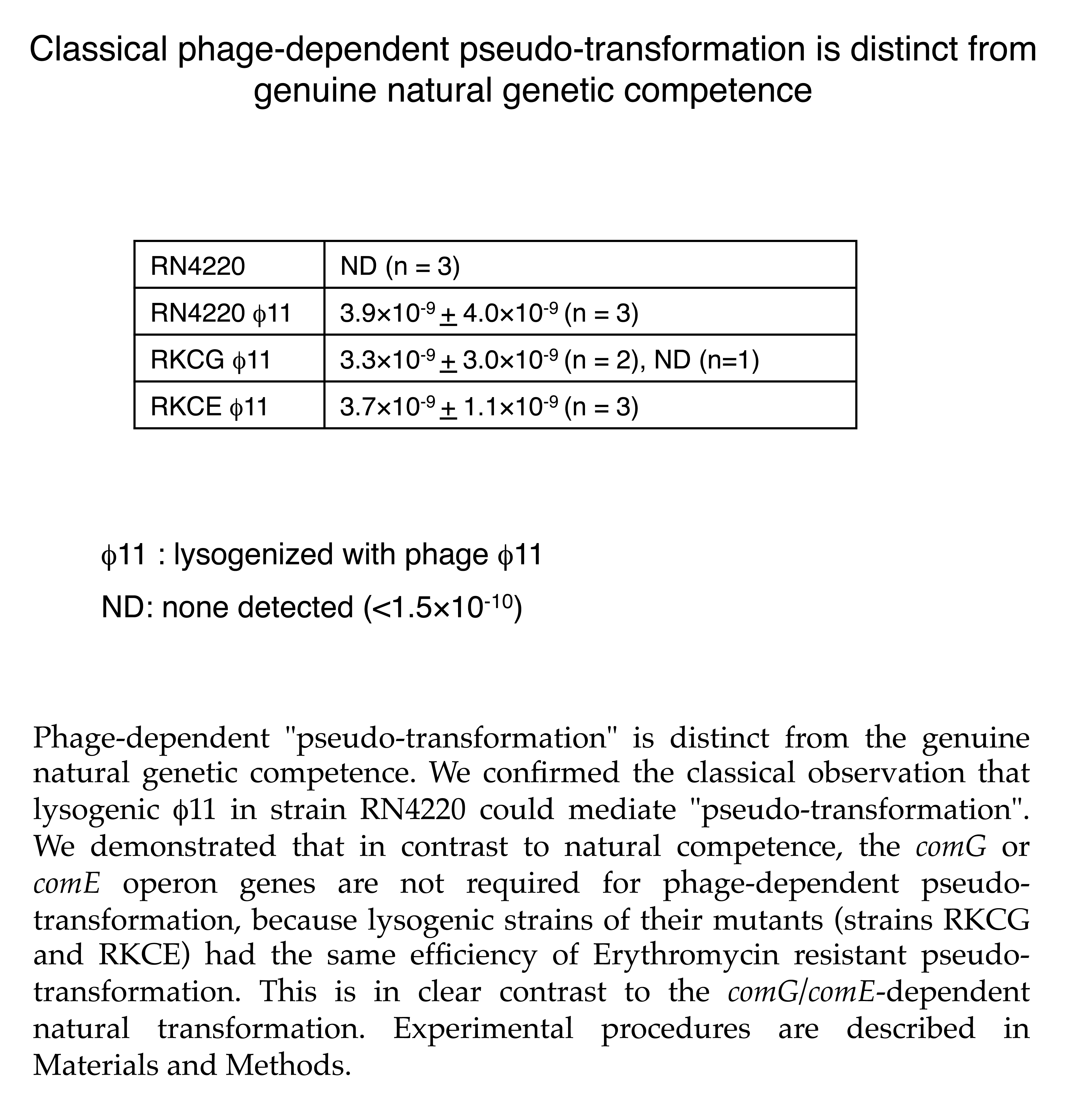

Supplement: Figure S7 — Phage-dependent “pseudo-transformation" in S . aureus does not require the comG and comE operons, unlike natural genetic competence. Experiments were carried out based on the method previously described [77] with some modifications. In brief, S. aureus cells were grown in TSB medium at 37°C overnight with shaking (180 rpm). Cells were recovered by centrifugation, washed once with 0.1 M Tris-malate (pH 7.0) and resuspended in 0.1 M Tris-malate (pH 7.0) supplemented with 0.1 M CaCl2. 24 µg of the N315 purified genomic DNA was added to 1 ml of cell suspension, and cells were incubated at room temperature for 40 min. Cells were recovered by centrifugation, suspended in drug-free BHI medium and incubated for 1 h at 37°C with shaking (100 rpm). Cells were mixed with BHI-agar medium pre-cooled to 55°C and supplemented with 5 µg/ml erythromycin and poured into plates. After two days incubation at 37°C, colonies were counted and checked for the presence of the erm gene by PCR with primers ErmA1 and ErmA2. (TIF) [file ppat.1003003.s007.tif]

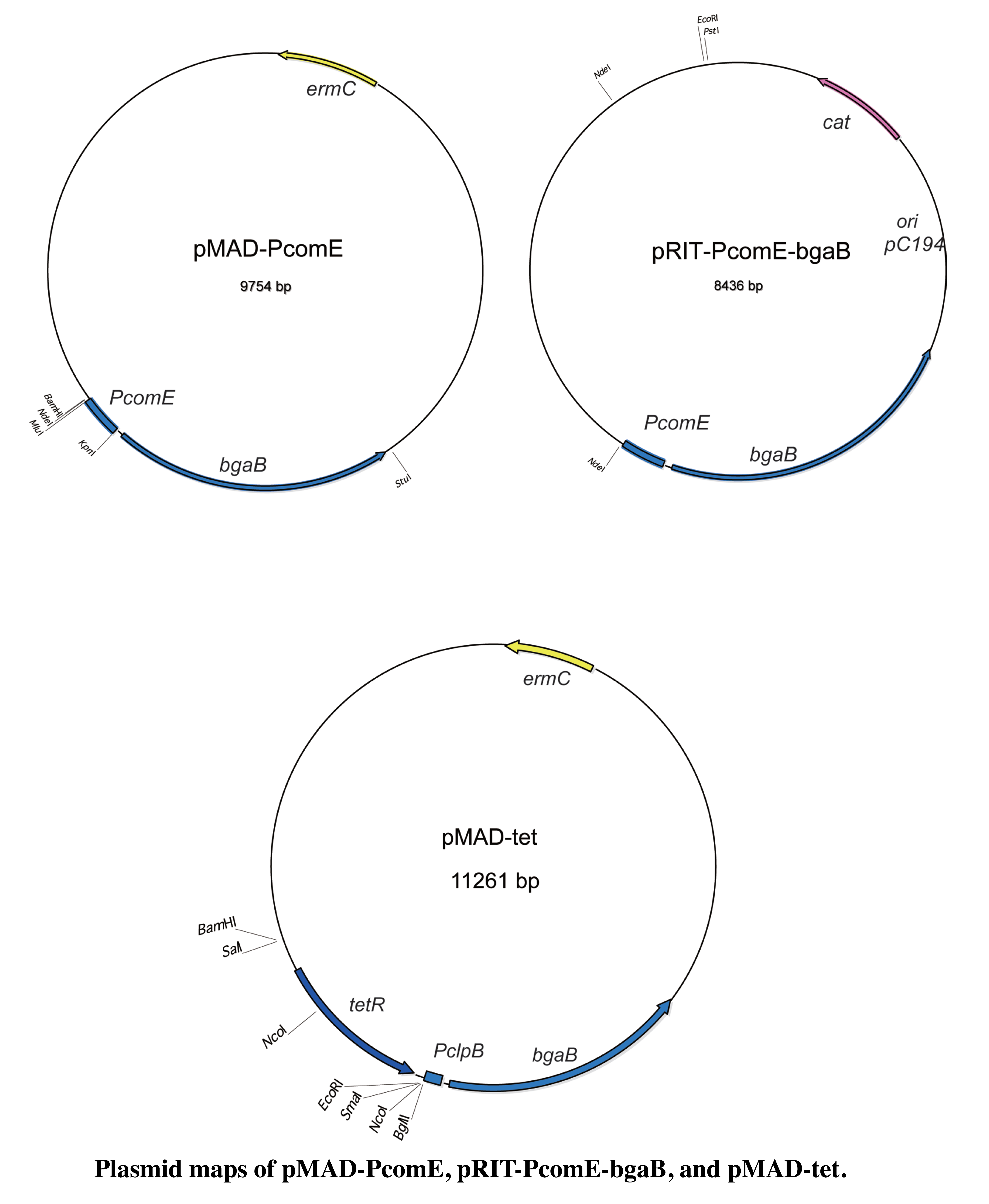

Supplement: Figure S8 — Plasmid maps of plasmids pMAD-PcomE, pRIT-PcomE-bgaB, and pMAD-tet. Plasmids pMAD-PcomE and pRIT-PcomE-bgaB are intermediates used for constructing plasmid pTet-rep. Plasmid pRIT-PcomE-bgaB was used as a reporter to follow SigH activity in Figure 1C. The Pspa promoter sequence is located between the NdeI and EcoRI sites, and hence does not affect expression of the PcomE-bgaB transcriptional fusion. Plasmid pMAD-tet was modified from pMAD for use in S. aureus strains resistant to erythromycin. (TIF) [file ppat.1003003.s008.tif]
