# Supplementary material for: Expression of a Cryptic Secondary Sigma Factor Gene Unveils Natural Competence for DNA Transformation in Staphylococcus aureus
Source: PLoS Pathog. 2012 Nov 1;8(11):e1003003. doi: 10.1371/journal.ppat.1003003 (PMC3486894; doi:10.1371/journal.ppat.1003003)
Supplement: Table S1 — Composition of CS2 medium. (PDF) [file ppat.1003003.s009.pdf]

Table S1 Composition of CS2 medium

	stock conc.	final conc.	1000 ml
**Sterile MilliQ water**			710ml
**Solution A**			100ml
Na_2HPO_4	71.4 g/L		
KH_2PO_4	30 g/L		
$(NH_4)_2SO_4$	20 g/L		
**Solution B-1**			10 ml
$MgSO_4 \cdot 7H_2O$	5 g/L	50 mg/L	
$MnSO_4 \cdot 5H_2O$	0.5418 g/L	5.4 mg/L	
**Solution B-2**			10 ml
$FeSO_4 \cdot 7H_2O$	0.28 g/L	2.8 mg/L	
**Solution C**			25 ml
40% Glucose	40%(w/v)	1%	
**Solution D**			10 ml
Biotin	10 mg/L	0.1 mg/L	
Nicotinic acid	200 mg/L	2 mg/L	
D-Panthothenic acid	200 mg/L	2 mg/L	
Pyridoxyne hydrochloride	400 mg/L	4 mg/L	
Riboflavin	200 mg/L	2 mg/L	
Thiamine hydrochloride	200 mg/L	2 mg/L	
**Adenine Solution**			50 ml
Adenine $\cdot$ $1/2H_2SO_4$	300 mg/L	15mg/L	
**Guanine Solution***			50 ml
Guanine	592 mg/L	30mg/L	
**Trace elements Solution**			10 ml
$CaCl_2$	109.94 mg/L	**	
1M $ZnSO_4$	105.2 ul	0.17mg/L	
100mM $CuSO_4$	504 ul	0.08mg/L	
$CoCl_2 \cdot 6H_2O$	12 mg/L	0.12mg/L	
$Na_2MoO_4 \cdot 2H_2O$	12 mg/L	0.12mg/L	
**Amino Acids Solution** ***			20 ml
**Glutamine Solution**			10 ml
L-Glutamine	29.2 g/L	0.29 g/L	
**Solution Ca**			0.14
$CaCl_2$	55.4987 g/L	8.9mg/L**	

* in 0.05N NaOH

** total

*** RPMI1640 amino acids solution (50X) (Sigma, R7131)
